# Phenolic Thiazoles with Antioxidant and Antiradical Activity. Synthesis, In Vitro Evaluation, Toxicity, Electrochemical Behavior, Quantum Studies and Antimicrobial Screening

**DOI:** 10.3390/antiox10111707

**Published:** 2021-10-27

**Authors:** Gabriel Marc, Anca Stana, Ana Horiana Franchini, Dan Cristian Vodnar, Gabriel Barta, Mihaela Tertiş, Iulia Şanta, Cecilia Cristea, Adrian Pîrnău, Alexandra Ciorîţă, Bogdan Dume, Vlad-Alexandru Toma, Laurian Vlase, Ilioara Oniga, Ovidiu Oniga

**Affiliations:** 1 Department of Pharmaceutical Chemistry, “Iuliu Hațieganu” University of Medicine and Pharmacy, 41 Victor Babeș Street, RO-400012 Cluj-Napoca, Romania; marc.gabriel@umfcluj.ro (G.M.); ana.ho.franchini@elearn.umfcluj.ro (A.H.F.); ooniga@umfcluj.ro (O.O.); 2 Department of Food Science and Technology, University of Agricultural Sciences and Veterinary Medicine, 3-5 Manăştur Street, RO-400372 Cluj-Napoca, Romania; gabriel.barta@usamvcluj.ro; 3 Department of Analytical Chemistry, Faculty of Pharmacy, “Iuliu Haţieganu” University of Medicine and Pharmacy, 4 Louis Pasteur Street, RO-400349 Cluj-Napoca, Romania; mihaela.tertis@umfcluj.ro (M.T.); iulia.nico.santa@elearn.umfcluj.ro (I.Ş.); ccristea@umfcluj.ro (C.C.); 4 National Institute for Research and Development of Isotopic and Molecular Technologies, 67-103 Donath Street, RO-400293 Cluj-Napoca, Romania; apirnau@itim-cj.ro (A.P.); alexandra.ciorita@itim-cj.ro (A.C.); vlad.toma@ubbcluj.ro (V.-A.T.); 5 Department of Molecular Biology and Biotechnologies, Babeș-Bolyai University, 4-6 Clinicilor Street, RO-400006 Cluj-Napoca, Romania; bogdan.dume@stud.ubbcluj.ro; 6 NIRDBS Bucharest, Institute of Biological Research, 48 Republicii Street, RO-400015 Cluj-Napoca, Romania; 7 Department of Pharmaceutical Technology and Biopharmaceutics, “Iuliu Hațieganu” University of Medicine and Pharmacy, 41 Victor Babeș Street, RO-400012 Cluj-Napoca, Romania; laurian.vlase@umfcluj.ro; 8 Department of Pharmacognosy, “Iuliu Haţieganu” University of Medicine and Pharmacy, 12 Ion Creangă Street, RO-400010 Cluj-Napoca, Romania; ioniga@umfcluj.ro

**Keywords:** polyphenols, thiazole, antioxidant activity, antiradical activity, synthesis, cytotoxicity, antimicrobial activity, quantum studies, electrochemical behavior

## Abstract

Oxidative stress represents the underlying cause of many chronic diseases in human; therefore, the development of potent antioxidant compounds for preventing or treating such conditions is useful. Starting from the good antioxidant and antiradical properties identified for the previously reported Dihydroxy-Phenyl-Thiazol-Hydrazinium chloride (DPTH), we synthesized a congeneric series of phenolic thiazoles. The radical scavenging activity, and the antioxidant and chelation potential were assessed in vitro, a series of quantum descriptors were calculated, and the electrochemical behavior of the synthesized compounds was studied to evaluate the impact on the antioxidant and antiradical activities. In addition, their antibacterial and antifungal properties were evaluated against seven aerobic bacterial strains and a strain of *C. albicans*, and their cytotoxicity was assessed in vitro. Compounds **5a-b**, **7a-b** and **8a-b** presented remarkable antioxidant and antiradical properties, and compounds **5a-b**, **7a** and **8a** displayed good Cu^+2^ chelating activity. Compounds **7a** and **8a** were very active against *P. aeruginosa* ATCC 27853 compared to norfloxacin, and proved less cytotoxic than ascorbic acid against the human keratinocyte cell line (HaCaT cells, CLS-300493). Several phenolic compounds from the synthesized series presented excellent antioxidant activity and notable anti-*Pseudomonas* potential.

## 1. Introduction

Oxidation is a necessary physiological process taking place in all living organisms. However, an imbalance between the generation and build-up of reactive oxygen and nitrogen species (ROS and RNS) and the capacity of a biological system to neutralize these reactive radicals leads to increasing oxidative stress, a phenomenon that is thought to be the underlying cause for the pathogenesis of many chronic diseases in humans [1]. Although it is impossible to entirely avoid ROS and RNS exposure and oxidative stress, the protection against oxidative damage and chronic pathologies can be accomplished through various endogenous and exogenous antioxidants. Many natural or synthetic antioxidants (vitamin E, vitamin C, flavonoids, polyphenols, etc.) have been recently exploited due to their favorable effect against oxidative stress, which may prove useful for treating or preventing several human conditions caused by excessive oxidants and free radicals’ production [2].

The very good antioxidant and antiradical properties identified for the compound Dihydroxy-Phenyl-Thiazol-Hydrazinium chloride (DPTH), developed by our team and named in this paper **5a**, were the starting point for the present research [3].

**Figure 1:**
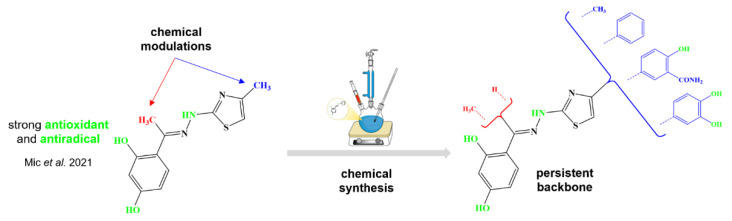
The hypothesis for the design of this research study.

The structure of compound **5a** (DPTH from our previous paper) includes a benzene ring substituted with two phenolic groups positioned at 1,3 and a thiazole ring connected to the benzene nucleus by a hydrazone moiety. The remarkable antioxidant and antiradical activity of the compound can be attributed to the two phenolic groups and/or the hydrazone moiety from its structure. The previously reported compound **5a** has two methyl groups—one on the azomethine carbon atom and the other in position 4 of the thiazole ring—which represented the sites of chemical modulations in our present study (Figure 1).

Encouraged by the results obtained in the in vitro evaluations of the antioxidant and antiradical properties of compound **5a**, we set out to develop a complete congeneric series of eight compounds, based on the two types of substitutions we proposed (2 × 4 elements of variability).

First, on the azomethine carbon atom, the compounds in the present study may have a methyl group (series **a** of compounds) or a hydrogen atom (series **b** of compounds). Therefore, we consider that this structural modulation is able to influence the antioxidant and antiradical activity of the compounds, because the hydrazone function was identified in a previous report as having significant antioxidant and antiradical activity due to the N-H group [4].

The second structural modulation we propose is in position 4 of the thiazole ring. One mode of substitution is with a methyl group, which is electron donating (compounds **5a-b**); the second is with a phenyl ring, which is electron withdrawing (compounds **6a-b**); and the third is with a phenyl ring substituted with phenolic groups (compounds **7a-b** and **8a-b**).

It is known from the literature that the conjugation of electrons is able to ensure a high stability of the structure resulting from a compound with antiradical properties following the neutralization of an external radical [5,6,7].

We proposed to perform these structural modulations because the backbone of the compounds allows multicenter electronic conjugation, so the effect of a substitution can be significantly manifested, not only on an antioxidant and/or antiradical group in its vicinity, but it can also influence the behavior of antioxidant and/or antiradical distal groups due to conjugation.

Therefore, it was possible to evaluate the manner in which the antioxidant and antiradical activity of the compounds is influenced by the proposed substitutions.

The antioxidant and antiradical properties of the newly synthesized molecules were evaluated using several antiradical, electron transfer, and ferrous and cupric ions chelation assays. Moreover, a series of molecular quantum descriptors were determined in silico in order to assess their influence on the antioxidant and antiradical activities of the synthesized compounds, and their electrochemical behavior in terms of antioxidant character was studied.

The literature includes reports of researchers who synthesized compounds with a benzylidene-2-hydrazinyl thiazole structure, who made a number of interesting observations: compounds that have a nitrogen-type substituent (i.e., pyridine) or an OH group in the *ortho* position of the benzylidene moiety, have good antimicrobial or antimalarial activity compared to compounds that have the polar substituent elsewhere on the aromatic nucleus [8,9,10]. As phenolic compounds have been reported to possess good antimicrobial properties [11,12], we also evaluated the antibacterial and antifungal activity of the synthesized compounds. 

In addition, their cytotoxicity was evaluated in vitro on a human keratinocyte cell line and their effect on the cells integrity was studied, in order to establish their safety for use in further biological activity investigations.

## 2. Materials and Methods

### 2.1. Chemistry

All chemicals and reagents used in the present research had appropriate grade purity and were purchased from local suppliers. The melting points were measured using an MPM-H1 melting point device (Schorpp Gerätetechnik, Überlingen, Germany), using the glass capillary method. The MS spectra of the compounds were recorded on an Agilent 1100 series device, in negative ionization mode, connected to an Agilent Ion Trap SL mass spectrometer (Agilent Technologies, Santa Clara, CA, USA). The IR spectra were recorded under vacuum, using a FT/IR 6100 spectrometer (Jasco, Cremella, Italy) in KBr pellets. The ^1^H-NMR and ^13^C-NMR spectra were recorded using an Avance NMR spectrometer (Bruker, Karlsruhe, Germany) in dimethyl sulfoxide-d_6_ (DMSO-d_6_). Chemical shift values were reported in δ units, using tetramethylsilane as an internal standard.

**Scheme 1:**
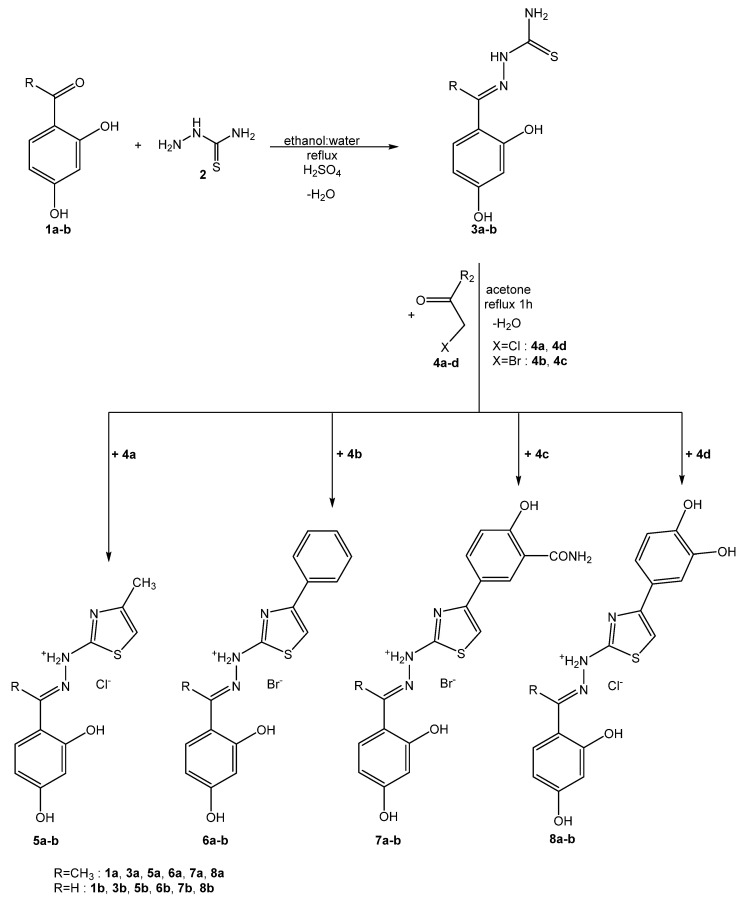
The general route for the synthesis of target compounds.

The route followed for synthesizing the intermediate and final compounds is presented in Scheme 1. The synthesis of intermediate compounds **3a** and **3b** and that of final compound **5a** were previously reported in the literature [3,13,14]. To a methanol solution (60 mL) of 2,4-dihydroxybenzaldehyde **1b** or 2,4-dihydroxyacetophenone **1a** (20 mmol) was added a hot aqueous solution (30 mL) of thiosemicarbazide **2** (20 mmol) and to the resulted mixtures was added a drop of concentrated H_2_SO_4_. The reaction mixtures were refluxed for 2 h and afterwards they were cooled to room temperature. The resulting precipitates (compounds **3a** and **3b**) were filtered off and vacuum dried. The synthesis of all final compounds was performed according to a method already described in the literature [3,15]. A quantity of 10 mmol of compound **3a-b** was mixed with 70 mL of hot anhydrous acetone in a round bottom glass flask under magnetic stirring until solubilization, then 10 mmol of the corresponding compound **4a-d** was added. Afterwards, the mixture was refluxed for one hour in a steam bath. After reaction completion was confirmed by TLC, the reaction mixture was left with continuous stirring to cool in order to allow the total precipitation of the desired final product. The precipitate was filtered using vacuum and crystallized two times from anhydrous acetone (2 × 50 mL), to obtain the final compounds **5b**, **6a-b**, **7a-b**, and **8a-b**.

The compounds’ purity was initially confirmed by thin layer chromatography (TLC) and afterwards by high performance liquid chromatography (HPLC). (E)-2-(2,4-dihydroxybenzylidene)-1-(4-methylthiazol-2-yl)hydrazin-1-ium chloride (**5b**): white solid; carbonization over 240 °C; yield = 71%; FT IR (KBr) ν_max_ cm^−1^: 3425 (OH), 3197 (NH), 3100, 2831, 1627, 1614, 1520 (C=C, C=N), 1361, 1217 (OH), 971; MS: *m/z* = 248.1; ^1^H-NMR (DMSO-d_6_, 500 MHz) δ: 2.236 (s, 3H, -CH_3_), 6.358 (dd, 1H, Ar, *J* = 8.5 Hz, *J* = 2.5 Hz), 6.404 (dd, 1H, Ar, *J* = 2 Hz), 6.584 (s, 1H, Th-C5), 7.560 (br, 1H, Ar), 8.488 (s, 1H, -CH=); ^13^C-NMR (DMSO-d_6_, 125 MHz) δ: 14.850 (-CH_3_), 100.962 (Th-C5), 102.943 (Ar), 108.591 (Ar), 111.293 (Ar), 128.868 (Ar), 149.110 (-CH=), 159.182 (Ar-OH), 161.807 (Ar-OH), 167.329 (Th-C2).

(E)-2-(1-(2,4-dihydroxyphenyl)ethylidene)-1-(4-phenylthiazol-2-yl)hydrazin-1-ium bromide (**6a**): pale yellow solid; carbonization over 264 °C; yield = 89%; FT IR (KBr) ν_max_ cm^−1^: 3425 (OH), 3098 (NH), 1620, 1567, 1520 (C=C, C=N), 1366, 1329, 1269, 1149 (OH), 986; MS: *m/z* = 325.2; ^1^H-NMR (DMSO-d_6_, 500 MHz) δ: 2.393 (s, 3H, -CH_3_), 6.317 (d, 1H, Ar, *J* = 2.5 Hz), 6.359 (dd, 1H, Ar, *J* = 9 Hz, *J* = 2.5 Hz), 7.277 (s, 1H, Th-C5), 7.333 (t, 1H, Ar, *J* = 7.5 Hz), 7.407-7.447 (m, 3H, Ar), 7.886 (d, 2H, Ar, *J* = 7 Hz); ^13^C-NMR (DMSO-d_6_, 125 MHz) δ: 14.878 (-CH_3_), 103.468 (Ar), 107.597 (Ar), 112.490 (Ar), 126.062 (Ar), 128.357 (Ar), 129.062 (Ar), 130.044 (Ar), 159.882 (Ar-OH), 160.288 (Ar-OH), 168.225 (Th-C2).

(E)-2-(2,4-dihydroxybenzylidene)-1-(4-phenylthiazol-2-yl)hydrazin-1-ium bromide (**6b**): pale yellow solid; carbonization over 260 °C; yield = 75%; FT IR (KBr) ν_max_ cm^−1^: 3394 (OH), 3287 (NH), 3066, 2923, 2831, 1632, 1616, 1579, 1564 (C=C, C=N), 1512, 1502, 1415, 1315, 1241, 1229, 1165 (OH), 1103, 980, 743; MS: *m/z* = 310.3; ^1^H-NMR (DMSO-d_6_, 500 MHz) δ: 6.349-6.381 (m, 2H, Ar), 7.302 (s, 1H, Th-C5), 7.422-7.471 (m, 3H, Ar), 7.348 (t, 1H, Ar, *J* = 7.5 Hz), 7.825 (d, 2H, Ar, *J* = 8.5 Hz), 8.38 (s, 1H, -CH=); ^13^C-NMR (DMSO-d_6_, 125 MHz) δ: 102.985 (Th-C5), 103.734 (Ar), 108.493 (Ar), 111.790 (Ar), 126.292 (Ar), 128.588 (Ar), 129.169 (Ar), 133.537 (Ar), 143.665 (-CH=), 158.517 (Ar-OH), 160.981 (Ar-OH), 168.428 (Th-C2).

(E)-1-(4-(3-carbamoyl-4-hydroxyphenyl)thiazol-2-yl)-2-(1-(2,4-dihydroxyphenyl) ethylidene)hydrazin-1-ium bromide (**7a**): pale yellow solid; carbonization over 290 °C; yield = 78%; FT IR (KBr) ν_max_ cm^−1^: 3382 (OH), 3275 (OH), 3202 (NH), 3099, 2947, 2832, 1659, 1624, 1587, 1554 (C=C, C=N), 1518, 1442, 1368, 1215 (OH), 1126, 1007, 835; MS: *m/z* = 383.4; ^1^H-NMR (DMSO-d_6_, 500 MHz) δ: 2.392 (s, 3H, -CH_3_), 6.315 (d, 1H, Ar, *J* = 2.5Hz), 6.360 (dd, 1H, Ar, *J* = 2.5 Hz, *J* = 8.5 Hz), 6.954 (d, 1H, Ar, *J* = 8.5 Hz), 7.115 (s, 1H, Th-C5), 7.415 (d, 1H, Ar, *J* = 9 Hz), 7.893 (dd, 1H, Ar, *J* = 2.5 Hz, *J* = 8.5 Hz), 7.967 (br, 1H, -NH), 8.358 (s, 1H, Ar), 8.449 (br, 1H, -NH); ^13^C-NMR (DMSO-d_6_, 125 MHz) δ: 14.906 (-CH_3_), 103.454 (Ar), 107.618 (Ar), 112.483 (Ar), 115.192 (Ar), 118.033 (Ar-CONH_2_), 126.118 (Ar), 130.072 (Ar), 131.808 (Ar), 159.903 (Ar-OH), 160.316 (Ar-OH), 160.981 (Ar-OH), 166.267 (Th-C2), 172.152 (-CONH_2_).

(E)-1-(4-(3-carbamoyl-4-hydroxyphenyl)thiazol-2-yl)-2-(2,4-dihydroxybenzylidene) hydrazin-1-ium bromide (**7b**): pale yellow solid; carbonization over 260 °C; yield = 71%; FT IR (KBr) ν_max_ cm^−1^: 3397 (OH), 3283 (NH), 3198, 3134, 2831, 1673, 1629, 1611, 1584 (C=C, C=N), 1367, 1217 (OH), 1119, 1005, 977, 774; MS: *m/z* = 369.4; ^1^H-NMR (DMSO-d_6_, 500 MHz) δ: 6.337-6.360 (m, 2H, Ar), 6.941 (d, 1H, Ar, *J* = 9 Hz), 7.124 (s, 1H, Th-C5), 7.422 (d, 1H, Ar, *J* = 9 Hz), 7.866 (dd, 1H, Ar, *J* = 8.5 Hz, *J* = 2 Hz), 7.942 (br, 1H, -NH), 8.262 (s, 1H, -CH= ), 8.232 (d, 1H, Ar, *J* = 2 Hz), 8.480 (br, 1H, -NH); ^13^C-NMR (DMSO-d_6_, 125 MHz) δ: 101.473 (Th-C5), 102.978 (Ar), 108.402 (Ar), 111.937 (Ar), 115.192 (Ar), 117.956 (Ar), 126.125 (Ar), 128.973 (Ar), 131.906 (Ar), 142.776 (-CH=), 158.349 (Ar-OH), 160.673 (Ar-OH), 160.869 (Ar-OH), 168.414 (Th-C2), 172.201 (-CONH_2_).

(E)-2-(1-(2,4-dihydroxyphenyl)ethylidene)-1-(4-(3,4-dihydroxyphenyl)thiazol-2-yl)hydrazin-1-ium chloride (**8a**): yellow solid; carbonization over 260 °C; yield = 83%; FT IR (KBr) ν_max_ cm^−1^: 3401 (OH), 3184 (NH), 3163, 1615, 1600, 1516 (C=C, C=N), 1359, 1282, 1238 (OH), 1198, 1178, 1112, 847, 808, 618; MS: *m/z* = 356.5; ^1^H-NMR (DMSO-d_6_, 500 MHz) δ: 2.394 (s, 3H, -CH_3_), 6.318 (d, 1H, Ar, *J* = 2.5 Hz), 6.359 (dd, 1H, Ar, *J* = 2.5 Hz, *J* = 8.5 Hz), 6.781 (d, 1H, Ar, *J* = 8 Hz), 6.879 (s, 1H, Th-C5), 7.122 (dd, 1H, Ar, *J* = 2 Hz, *J* = 8 Hz), 7.218 (d, 1H, Ar, *J* = 2 Hz); ^13^C-NMR (DMSO-d_6_, 125 MHz) δ: 14.969 (-CH_3_), 103.447 (Ar), 107.597 (Ar), 112.525 (Ar), 113.834 (Ar), 116.165 (Ar), 117.634 (Ar), 127.958 (Ar), 130.044 (Ar), 145.771 (Ar-OH), 146.233 (Ar-OH), 159.917 (Ar-OH), 160.323 (Ar-OH), 168.085 (Th-C2).

(E)-2-(2,4-dihydroxybenzylidene)-1-(4-(3,4-dihydroxyphenyl)thiazol-2-yl)hydrazin-1-ium chloride (**8b**): orange solid; carbonization over 260 °C; yield = 74%; FT IR (KBr) ν_max_ cm^−1^: 3379 (OH), 3112 (NH), 1639, 1623, 1610, 1532, 1523 (C=C, C=N), 1336, 1303, 1278 (OH), 1240, 1222, 849; MS: *m/z* = 342.5; ^1^H-NMR (DMSO-d_6_, 500 MHz) δ: 6.358 (dd, 1H, Ar, *J* = 2.5 Hz, *J* = 8 Hz), 6.391 (d, 1H, Ar, *J* = 2.5 Hz), 6.978 (d, 1H, Ar, *J* = 8 Hz), 6.957 (s, 1H, Th-C5), 7.102 (dd, 1H, Ar, *J* = 2 Hz, *J* = 8 Hz), 7.210 (d, 1H, Ar, *J* = 2 Hz), 7.455 (d, 1H, Ar, *J* = 8 Hz), 8.362 (s, 1H, -CH=); ^13^C-NMR (DMSO-d_6_, 125 MHz) δ: 100.647 (Th-C5), 102.999 (Ar), 108.486 (Ar), 111.685 (Ar), 114.072 (Ar), 116.221 (Ar), 117.879 (Ar), 128.896 (Ar), 145.778 (Ar-OH), 146.401 (Ar-OH), 158.650 (Ar-OH), 161.100 (Ar-OH), 168.113 (Th-C2).

### 2.2. In Vitro Antioxidant, Antiradical and Chelation Assays

The stock solutions (1 mg/mL) of the reference compounds and of the tested compounds were prepared in dimethyl sulfoxide (DMSO). Supplementary dilutions in DMSO of the initial stock solutions were made, according to the needs of each assay.

Using an UV-VIS Jasco V-530 spectrophotometer (Jasco International Co., Tokyo, Japan), the in vitro assays were carried out using 10 mm single use plastic cuvettes. The compounds showed no absorption peaks near the wavelengths where the measurements were performed. All the assays were conducted in triplicate and the results are presented as averages.

#### 2.2.1. Antiradical Assays

##### ABTS˙^+^ Radical Scavenging Assay

The assay was based on the decolorization of green (2,2′-azinobis-(3-ethylbenzothiazoline-6-sulfonic acid) ABTS**˙**^+^ to ABTS, on the principle reported by Re et al. and was carried out according to our previously published research [16,17]. 

Quantities of 10, 15, 20, 30, and 50 µL from the 0.1 mg/mL stock solutions in DMSO of the compounds and ascorbic acid, used as standard, were poured into cuvettes and the appropriate volume of DMSO was added until a total volume of 50 µL was found in all cuvettes. Later, 1950 µL of ABTS**˙**^+^ reagent was added to all cuvettes and they were shaken for well over 10 min in the dark at room temperature. The absorbance of the resulted solutions found in cuvettes was determined spectrophotometrically at λ = 734 nm. The ABTS scavenging activity of the compounds was calculated using the following Equation (1):

(1) $$ABTS\;scavenging\;\left(\%\right)=\frac{control\;absorbance-sample\;absorbance}{control\;absorbance}\times 100$$

##### DPPH˙ Radical Scavenging Assay

The 2,2-diphenyl-1-picrylhydrazyl (DPPH**˙)** radical scavenging assay is based on the transfer of one hydrogen from the analyzed compound to the violet radical DPPH**˙**. This will convert it to a yellow compound, the change in absorbance being proportional to the amount of DPPH**˙** neutralized, according to Brand-Williams et al. [18,19].

The preparation of the DPPH**˙** reagent was previously reported by our group [17]. To all cuvettes were added 10, 15, 20, 30, and 50 µL from the 0.2 mg/mL stock solution of the analyzed compounds in DMSO, and trolox and ascorbic acid as reference compounds. Over the previous quantities, DMSO was added until a total volume of 50 µL was obtained in all cuvettes, and 1950 µL of DPPH**˙** solution was added to all cuvettes; then, they were shaken for well over 10 min in the dark at room temperature. The absorbances of the solutions were measured at λ = 517 nm. The DPPH**˙** radical scavenging activity of evaluated compounds was calculated using Equation (2):

(2) $$DPPH\;scavenging\;\left(\%\right)=\frac{control\;absorbance-sample\;absorbance}{control\;absorbance}\times 100$$

##### NO˙ Radical Scavenging Assay

Scavenging of the NO˙ radical was performed according to some adapted protocols reported in the literature, based on the nitroprusside decomposition at physiological pH and colorimetric quantification of the resulted products using the Griess reaction [20,21,22].

Over 200 µL of 10 mM sodium nitroprusside, 100 µL of test solutions (1 mg/mL in DMSO), and 400 µL phosphate-buffered saline (PBS, pH = 7.4) were added. The mixture was then incubated at room temperature for 150 min on a rotating shaker (GFL Gesellschaft für Labortechnik, Burgwedel, Germany). After that, 1 mL of sulfanilic acid 0.33% in 20% acetic acid was added and after 5 min, 1 mL of naphthylethylenediamine dichloride 0.1% was added to all test tubes. After 30 min, the absorbance of the sample, given by the chromophore (azo dye) formed during the diazotization of nitrite ions resulting from decomposition of sodium nitroprusside with sulfanilic acid and coupled with naphthylethylenediamine, was measured at 546 nm. The NO˙ radical scavenging activity of the tested compounds was calculated using Equation (3):

(3) $$NO˙\;scavenging\;\left(\%\right)=\frac{control\;absorbance-sample\;absorbance}{control\;absorbance}\times 100$$

#### 2.2.2. Electron Transfer Assays

##### Ferric Reducing Antioxidant Potential (FRAP) Assay

The reducing power of the tested compounds was determined using the FRAP assay, according to a modified method proposed initially by Benzie and Strain, which was previously reported by our group [3,17,19,23]. The reducing power of compounds was calculated as percent of the reference compounds, using Equation (4):

(4) $$\%\;of\;control\;activity=\frac{sample\;absorbance}{reference\;absorbance}\times 100$$

##### Phosphomolybdate Assay for Total Antioxidant Capacity (TAC) 

To determine the TAC of the tested compounds we used a procedure previously reported in the literature by our group [3,17,22,24]. The TAC of compounds was calculated using Equation (5):

(5) $$\%\;of\;control\;activity=\frac{sample\;absorbance}{reference\;absorbance}\times 100$$

##### Reducing Power (RP) Assay 

The followed protocol to assess the RP of compounds was previously reported by our group [3,17,22,25]. The RP of compounds was calculated using Equation (6):

(6) $$\%\;of\;control\;activity=\frac{sample\;absorbance}{reference\;absorbance}\times 100$$

#### 2.2.3. Ferrous and Cupric Chelation Assays

##### Fe^2+^ Chelation Assay

The protocol used for assessing Fe^2+^ chelating capacity of the compounds was adapted from a report of Benzie and Strain and previously reported by our group [3,23,26]. The iron(II) chelation ability of compounds was calculated using Equation (7):

(7) $$iron\;chelation\;\left(\%\right)=\frac{control\;absorbance-sample\;absorbance}{control\;absorbance}\times 100$$

##### Cu^2+^ Chelation Assay

The protocol used for evaluating Cu^2+^ chelating activity of the compounds and the disodium salt of ethylenediaminetetraacetic acid (EDTA-Na_2_) was performed using the method presented by Wu et al. with some modifications [27,28]. For each compound, 100, 200, 300, 400, and 500 µL were taken from the stock solutions (1 mg/mL in DMSO) and DMSO was added, in order to obtain a total volume of 500 µL in each test tube. Over them, 400 µL of 3 mM CuSO_4_ solution in hexamine buffer (10 mM hexamine and 10 mM KCl) was added. After energic shaking for over 5 min, 75 µL of murexide 1 mM and 2 mL of water were added, and after 3 min of incubation at room temperature, the absorbance was read at 485 and 520 nm. The two wavelengths correspond to the absorbance of murexide-copper(II) complex and free murexide, respectively. The ratio between the two absorbances are proportional to the free copper(II) in solution. The copper chelation capacity of the compounds was calculated using Equation (8):

(8) $$copper\;chelation\;\left(\%\right)=\frac{{\left(\frac{A_{485}}{A_{520}}\right)}_{control}-{\left(\frac{A_{485}}{A_{520}}\right)}_{sample}}{{\left(\frac{A_{485}}{A_{520}}\right)}_{control}}\times 100$$

### 2.3. Electrochemical Behavior of Compounds

The evaluated compounds’ stock solutions used were prepared at a concentration of 5 mM. To test their redox properties, dilutions of 0.5 mM and 2 mM were prepared in 0.1 M HClO_4_ electrolyte (pH = 1.2). Screen-printed electrodes (SPEs; Metrohm DropSens, Oviedo, Spain) (planar electrodes) with graphite and gold working electrodes were used. Cyclic voltammetry (CV) and differential pulse voltammetry (DPV) were the electrochemical methods applied. The experimental conditions for each test are mentioned near the corresponding data (Appendix A).

The antioxidant properties were evaluated in terms of their activity against some free radicals, and other compounds reported in the literature, relevant for this purpose, such as:

- ferric ions (Fe^3+^)—used to test the reduction of the antioxidant character of the compounds;
- DPPH˙ free radical– used to test the scavenging effect of the compounds on this radical;
- hydrogen peroxide (H_2_O_2_)—used to test the scavenging effect of the synthesized molecules on this compound;
- 2,2,6,6-tetramethylpiperidinyl-1-oxy (TEMPO)—used to test the scavenging effect of the synthesized molecules on this compound.

### 2.4. Theoretical Quantum Calculations

Taking into account the structure of the compounds and the most common quantum parameters used to be correlated with the antioxidant and antiradical activity of the compounds, we were interested in calculating the energy of the frontier orbitals and the strength of the bonds that can be broken in order to release hydrogen atoms, also known as bond dissociation energy (BDE) [29,30,31].

In order to have a better view of the antioxidant and antiradical behavior of the compounds, and how this is influenced by the chemical substitutions made on the backbone of the compounds, some theoretical quantum parameters were calculated according to a previously reported procedure [4,17].

### 2.5. Antimicrobial Activity Evaluation

For evaluating the antimicrobial activity of the compounds, seven aerobic microbial strains were used: *S. aureus* ATCC 25923, *S. pyogenes* ATCC 19615, *P. aeruginosa* ATCC 27853, *S. enterica* ISM 83/37, *S. typhimurium* ATCC 14028, *E. coli* ATCC 8739, *E. coli* ATCC 25922, and *C. albicans* ATCC 102310 obtained from Food Biotechnology Laboratory, Life Sciences Institute, University of Agricultural Sciences, and Veterinary Medicine Cluj Napoca, where the experiment was performed. The antimicrobial activity of the compounds was determined using a modified microdilution technique, and the followed protocol was previously reported [32]. 

### 2.6. Cytotoxicity Studies

#### 2.6.1. Cytotoxicity Determination

To determine the cytotoxic action of the obtained compounds **5a-b**, **6a-b**, **7a-b**, and **8a-b**, a normal human keratinocytes cell line was used (HaCaT keratinocytes, CLS-300493, Eppelheim, Germany). HaCaT cells were cultured in Dulbecco’s Modified Eagle Media (DMEM) supplemented with 1% L-glutamine, 1% penicillin-streptomycin, and 5% fetal bovine serum. At 80% confluence, the cells were transferred to 96 well plates at a confluence of 12×10^3^ cells/well and left to attach for 24 h at 37°C and 5% CO_2_ in a humidified atmosphere. The selected compounds were tested in increasing concentrations with the 3-(4,5-dimethylthiazol-2-yl)-2,5-diphenyltetrazolium bromide (MTT) method, based on which the IC_50_ (the concentration at which 50% of the cells were affected) was calculated [33].

Each plate contained controls with culture media and compounds **5a-b**, **6a-b**, **7a-b**, and **8a-b** without cells under the same tested concentrations, untreated controls, and positive controls (cells treated with 2% Tween 20 and 0.3% H_2_O_2_), and one plate was incubated with ascorbic acid (AA) solution using the same concentration. After 24 h, the culture media was replaced with 100 µL of MTT solution at a final concentration of 5 µg/mL/well and left to interact with the cells for 2 h in the incubator. After this, the MTT solution was replaced with 100 µL of isopropanol, left to dissolve the formazan crystals for 5 min, and read at 550 and 630 nm using a BioTek Epoch plate reader (BioTek Instruments, Winooski, VT, USA) and Gen5 software (version 1.09).

The IC_50_ concentrations were calculated according to Equation (9) [34]:

(9) $$MC=\frac{y-c}{m}$$

where *MC* = median concentration, *y* = 50%, *c* = constant generated by the exponential fitting of the data, and *m* = the coefficient calculated from the exponential fit of the data.

#### 2.6.2. HaCaT Cells Integrity Evaluation

In order to evaluate the cellular effect of the compounds, a selection from the whole batch of compounds was made, according to the structure–toxicity relationship that could be observed from the cytotoxicity determination. Analyzing the IC_50_ values obtained, two structural observations were made: in position 4 of the thiazole ring of the compounds the phenyl substituent is more favorable to the methyl rest and is preferable for the compounds to be phenylethylidene derivatives (series **a** of compounds) rather than benzylidene derivatives (series **b** of compounds). Even if the **b** series of compounds presented some intermediate IC_50_ values for compounds **5b** (IC_50_ = 50.34 µg/mL) and **7b** (IC_50_ = 42.36 µg/mL), because of the high cytotoxicity of the compounds **6b** (IC_50_ < 0.50 µg/mL) and **8b** (IC_50_ = 8.54 µg/mL), the whole **b** series of compounds was considered to be promiscuous and unsafe due to cytotoxicity. This conclusion can be strengthened by the observations of previous reports that found 2-benzylidenehydrazineyl-thiazole derivatives to have significant cytotoxic activity [4,35]. Thus, compounds **6a**, **7a**, and **8a** were selected for evaluation of their influence on HaCaT cells integrity. 

First, an acridine orange staining of the HaCaT cells was performed to investigate morphological features of the cells (3 × 10^4^ cells/well) after treatment with the selected compounds **6a**, **7a**, and **8a** at IC_50_ concentration for 24 h. The cells were left to attach to 10 mm Ø glass slides for 24 h, at a confluence of 3 × 10^4^ cells/glass slide. The slides were incubated for 10 min in acridine orange 1 µM in PBS 0.01M, and then the slides were investigated with an epifluorescence microscope Optika 510LD1 with 5 MP CCD cameras at 525 nm (green filter).

Second, a biochemical assay was performed, where the lactate dehydrogenase (LDH) and catalase (CAT) activity were determined. From the culture media of the cells exposed to the compounds **6a**, **7a**, and **8a,** at the determined IC_50_ value, the determinations were performed by the kinetic method using an UV-Vis spectrophotometer according to previous procedures, at 24 h [36,37]. All results obtained are expressed as mean ± standard deviation (SD). Comparisons between multiple groups were made using one-way ANOVA followed by Bonferroni’s post-hoc test. *p* < 0.05 was considered statistically significant and was interpreted as follows: * *p* < 0.05, ** *p* < 0.01, *** *p* < 0.001 for comparison with H_2_O_2_ treatment and ^#^ *p* < 0.05, ^##^ *p* < 0.01, ^###^ *p* < 0.001 when the comparison was made with the AA treatment. Statistical analyses were undertaken using GraphPad Prism 5.

### 2.7. Molecular Properties with Influence on the Pharmacokinetics of Compounds

From the literature reports it is known that polyphenols have poor bioavailability and absorption. Bioavailability is a pharmacokinetic parameter dependent on many processes and influenced by molecular properties [38,39]. Using the Swiss ADME web tool, some molecular properties were calculated, such as octanol-water partition coefficient, topological polar surface area, gastrointestinal absorption after oral administration, and octanol-water partition coefficients, as part of Lipinski’s rule of five [40,41,42]. pKa of the compounds was assessed using MolGpka [43]. The calculation of the molecular properties was performed for each parent compound of the final compounds **5a-b**, **6a-b**, **7a-b**, and **8a-b**, without the corresponding hydrohalide acid.

## 3. Results and Discussion

### 3.1. Chemical Synthesis and Characterization of Compounds

The thiosemicarbazones **3a-b** derived from resorcinol were synthesized by condensation of the corresponding thiosemicarbazide **2** and 2,4-dihydroxyacetophenone (**1a**) or 2,4-dihydroxybenzaldehyde (**1b**) following literature procedures [13,14]. The hydrazinyl-thiazole phenolic derivatives **5a-b**, **6a-b**, **7a-b**, and **8a-b** were obtained via Hantzsch heterocyclisation of thiosemicarbazones **3a-b** with the corresponding α-haloketones **4a-d** (Scheme 1) following a previously reported method [3]. The final compounds were synthesized in the form of their hydrochloride or hydrobromide salts. Compounds **3a-b** and **5a** were previously reported in the literature [3,13,14].

All final synthesized compounds presented high melting points due to the fact that they were salts with hydro acids. 

The spectral data obtained for the synthesized compounds were consistent with the proposed structures. In the MS spectra of final compounds, the corresponding molecular peaks and the hydrazinyl-thiazole peak was found. In the IR spectra of all final compounds, broad signals were identified at about 3400 cm^−1^ due to the phenolic O-H stretching and O-H bending bands at 1250 cm^−1^. The N-H stretching bands appearing at 3100–3300 cm^−1^ and at 1520–1530 cm^−1^ were identified absorption signals due to the C=C and C=N stretching.

In the ^1^H-NMR spectra of the synthesized compounds, all protons’ expected signals were identified, with the corresponding multiplicity and the related coupling constants. The broad signals expected for the O-H and N-H protons in the range of 8.5–12 ppm were overlapped and difficult to assign to a specific group, and were integrated as a whole.

In the ^13^C-NMR spectra of the target compounds, the aromatic carbons bearing the phenol groups appeared at high values, between 145.771 and 161.807 ppm. The most de-shielded values were found for the amide carbon atom at 172.152–172.201 ppm in the ^13^C-NMR spectra of compounds **7a-b** and for the carbon atoms from position 2 of the thiazole ring at 166.267–168.428 ppm in the ^13^C-NMR spectra of all final compounds.

The spectra of all synthesized compounds are provided in Supplementary Materials (Figures S1–S28).

### 3.2. In Vitro Antioxidant, Antiradical and Chelation Assays

The antioxidant potential of the molecules was evaluated based on the mechanisms possible for this activity, reported in the literature: hydrogen atom transfer, electron transfer, or chelation of transition metals [44]. Some of the in vitro laboratory protocols followed for the presented assays were undertaken at a semi-microscale level, according to our group’s previous reports [3,17,25]. All measurements were carried out in triplicate and the results are presented as averages. 

#### 3.2.1. Antiradical Assays

The antiradical potential of compounds was determined spectrophotometrically as the capacity to scavenge ABTS˙^+^, DPPH˙ and NO˙ radicals.

##### ABTS˙^+^ Radical Scavenging Assay

The capacity of the tested compounds and ascorbic acid to reduce ABTS**˙**^+^ is presented in Table 1. According to the results obtained in this assay, all the synthesized compounds presented important ABTS˙^+^ radical scavenging properties, the most active compounds being **5a-b**, **7b**, and **8a-b**, with lower IC_50_ values than that of ascorbic acid, used as standard antioxidant.

##### DPPH˙ Radical Scavenging Assay

The antiradical potential of compounds in comparison to reference compounds, ascorbic acid, and trolox, was determined spectrophotometrically, and the results are shown in Table 2. As can be observed from Table 2, compounds **5a-b**, **7a-b**, and **8a-b** presented the best capacity to scavenge the DPPH˙ radical.

##### NO˙ Radical Scavenging Assay

The NO*˙* antiradical potential of compounds, gentisic acid, and trolox were evaluated spectrophotometrically, based on the Griess reaction and the obtained results are presented in Table 3. According to the results obtained in this assay, all compounds presented antiradical properties, compounds **5a-b** and **8a-b** displaying the best ability to scavenge the NO*˙* radical, superior to that of trolox, which was used as a reference.

#### 3.2.2. Electron Transfer Assays

The antioxidant capacity of the synthesized compounds calculated as capacity of donation of electrons was ascertained spectrophotometrically, based on the FRAP, TAC, and RP assays.

##### Ferric Reducing Antioxidant Potential (FRAP) Assay

In this assay, the evaluated compounds reduced the ferric ions to ferrous ions, yielding a blue-colored complex at pH = 3.6 with 2,4,6-tris(2-pyridyl)-*s*-triazine (TPTZ). The intensity of the blue color is related with the capacity of the compound to reduce the ferric ions. The results are presented in Table 4 and reveal that compounds **5a-b**, **7a-b**, and **8a-b** presented the best antioxidant potential.

##### Phosphomolybdate Assay for Total Antioxidant Capacity (TAC)

The tested compounds transferred one electron to reduce Mo^6+^ to Mo^5+^. The amount of the green phosphateMo^5+^ complex is proportional with the activity of the compounds; results are presented in Table 4. The current assay proved an excellent electron-donating capacity for all compounds, with the exception of compound **6a**.

##### Reducing Power (RP) Assay

The reduction of ferricyanide to ferrocyanide by the evaluated compounds yielded the Perls’ Prussian blue. The reducing power of the compounds is proportional to the absorbance of the resulted solutions. The results obtained in the RP assay are presented in Table 4. All the compounds proved noticeable reducing properties, and the best activity was registered for compounds **5a-b** and **8a-b**.

Overall, from all three electron transfer assays and all three antiradical assays it can be observed that, in general, the benzylidene derivatives from series **b** presented better electron donating capacity and ability to scavenge the ABTS˙^+^, DPPH˙, and NO˙ radicals than their corresponding phenylethylidene derivatives from series **a**.

The very good activity identified for compound **8b** can be structurally explained due to the lack of the methyl group on the azomethine (**b** series) and the presence of the catechol group, in agreement with literature reports that suggest the high activity of this type of compound is due to the internal stabilization of the resulted radicals from the catechol fragment by intramolecular hydrogen bonding [45,46,47].

#### 3.2.3. Ferrous and Cupric Ions Chelation Assays

##### Fe^2+^ Chelation Assay

The chelating potential for the ferrous ions of the new compounds was determined, based on their competition for Fe^2+^ with ferrozine. The decrease in the absorbance suggested that the ferrous ions were sequestered by the evaluated compounds. The results of the assay are shown in Table 5. The chelating potential of compounds of the ferrous ions was negligible compared to that of EDTA-Na_2_.

##### Cu^2+^ Chelation Assay

The potential of chelating cupric ions of the tested compounds was evaluated based on the competition for Cu^2+^ with murexide, and the results are presented in Table 6. The chelating activity of compounds for the cupric ions found in the current assay was significant for compounds **5a-b**, **7a**, and **8a**, compared to that of EDTA-Na_2_.

### 3.3. Electrochemical Behavior of Compounds

The results obtained from the study of the electrochemical behavior of the synthesized compounds in terms of antioxidant character are presented in Appendix A and suggest that **5a**, **8a**, **5b**, and **8b** may possess this property.

The antioxidant character of the compounds was then tested by four different methods:

- testing the reducing antioxidant power of ferric ions;
- the radical scavenging effect of the novel compounds;
- scavenging of hydrogen peroxide;
- scavenging of 2,2,6,6-tetramethylpiperidinyl-1-oxy (TEMPO).

It was demonstrated that low oxidation potentials values reflect the predisposition of a compound for electron donation, and thus for exhibiting significant antioxidant/ antiradical activity. Moreover, for close oxidation potential values, the antioxidant capacity will be higher for compounds with higher oxidation peak current intensity.

**Figure 2:**
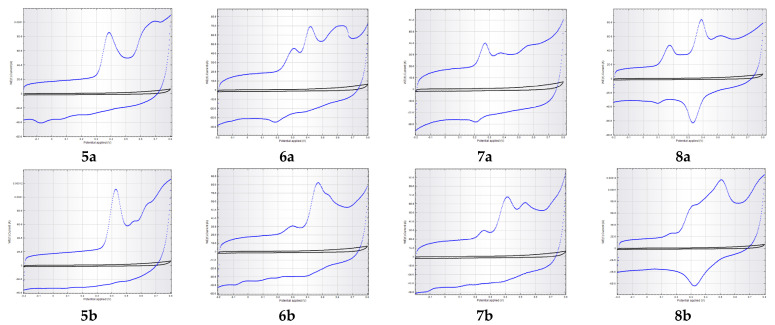
CVs for solutions of the novel synthesized compounds (blue) prepared in 0.1 M HClO_4_ on graphite-based SPE. Black line: CV recorded in 0.1 M HClO_4_.

**Figure 3:**
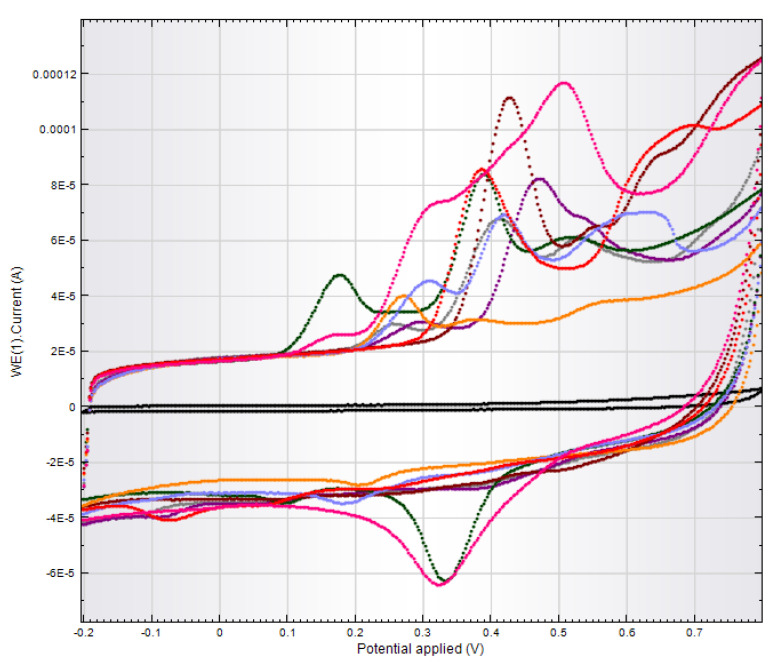
Overlapped CVs for solutions of the novel synthesized compounds: **5a**—red; **6a**—light blue; **7a**—orange; **8a**—green; **5b**—brown; **6b**—purple; **7b**—gray; **8b**—pink prepared in 0.1 M HClO_4_ on graphite-based SPE. Black line: CV recorded in 0.1 M HClO_4_.

In Figure 2, the cyclic voltammograms (CVs) for each of the compounds considered in this study, compared individually with the voltammogram obtained in the electrolyte (0.1 M HClO_4_), are presented. Thus, the oxidation/reduction peaks corresponding to the electrochemical transformations suffered by each compound can be observed in the potential range considered, in an acidic environment (low pH). From Figure 3, where the voltammograms of all eight compounds were presented together, a similarity is observed between **8a** and **8b**, and among the other compounds, whereas **5a** and **5b** can be noted as having the highest oxidation signals.

**Figure 4:**
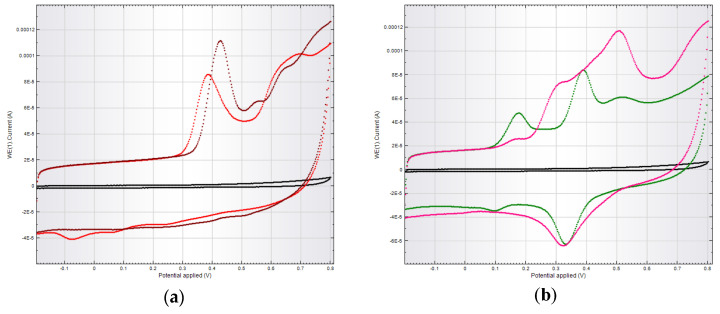
(**a**) Comparison between CVs for **5a** (red) and **5b** (brown); (**b**) comparison between CVs for **8a** (green) and **8b** (pink).

**5a** is represented together with **5b** (Figure 4a), and **8a** together with **8b** (Figure 4b).

When **5a** and **5b** are compared, it can be observed from Figure 4a and Table 7 that **5a** has the lowest oxidation potential for the main peak, which suggests that **5a** should have a higher antioxidant capacity than **5b**. In the case of **5b**, however, a more intense oxidation signal was obtained, so it can be concluded that these two compounds may have similar antioxidant capacity.

In the case of compounds **8a** and **8b**, there are signals of oxidation with high intensity, but which have reversible behavior, taking into account that they have a signal of intensity reduction close to a suitable reduction potential value (see the signals in Figure 4b and the corresponding values of the oxidation/reduction currents and potentials in Table 7). This reversible process occurs only in the case of **8a** and **8b**; both compounds are catechol derivatives, and observation is in agreement with previous literature reports [47].

The results obtained by all four methods correlated quite well and confirmed the antioxidant capacity of the four compounds **5a-b** and **8a-b**, which was in agreement with the results obtained in the in vitro antioxidant and antiradical assays.

### 3.4. Theoretical Quantum Calculations

The frontier molecular orbitals are very important in describing the potential chemical behavior of a compound, and were thus computed. The highest occupied molecular orbital (HOMO) indicates a good electron-donating capacity of the molecule and is linked to the susceptibility of the molecule to be attacked by electrophilic species. Alternatively, the energy of the lowest unoccupied molecular orbital (LUMO) is linked with its electron affinity and with the susceptibility to be attacked by a nucleophilic species. Reports in the literature suggest that the HOMO-LUMO gap may be a parameter that describes the antioxidant activity of the compounds [4,30].

HOMO and LUMO depicted for the compounds **5a-b, 6a-b, 7a-b** and **8a-b** are displayed in Table S1 (Supplementary Materials). Due to the substituent present in position 4 of the thiazole (methyl or phenyl), two means of distribution of the HOMO orbital could be identified. In the 4-methyl-thiazole derivatives **5a-b**, HOMO was found over the hydrazinyl-thiazole fragment, whereas in the 4-phenyl-thiazole derivatives **6a-b**, **7a-b** and **8a-b**, HOMO was found over the thiazolyl-4-phenyl fragment. The position of the HOMO on the specified regions of the molecules indicate that their redox behavior by extraction of electrons will take place due to involvement of that region. In the case of compounds **5a-b**, the increased presence of the HOMO over the hydrazinyl fragment and not over the diphenolic fragment is interesting, suggesting that the antioxidant and antiradical effect can be attributed less to the phenolic fragment and more to the hydrazinyl fragment. Substitution of the thiazole with a phenyl shifted the position of HOMO over the thiazolyl-4-phenyl fragment, reducing the influence of the hydrazine in the redox behavior of the compounds (**6a-b**, **7a-b**, and **8a-b)**. Therefore, the substitution with an aromatic nucleus in position 4 of the thiazole ring was essential for the redox behavior of the compounds that have it in their structure (**6a-b**, **7a-b**, and **8a-b)**.

Substitution of the azomethine carbon atom with methyl (**a** series of compounds) or hydrogen (**b** series of compounds) led to tiny changes in the positioning of HOMO between the two series of compounds. From a numerical point of view, in the case of HOMO, the difference between series **a** and series **b** of compounds was between 0.04 and 0.06 eV (Table 8), and larger differences appeared in the case of LUMO, up to 0.25 eV (**5a** vs. **5b**).

Interestingly, on the 2,4-diphenolic fragment HOMO was little present (**5a-b**) or even absent (**6a-b**, **7a-b** and **8a-b**), indicating that this fragment is not the one that causes the main antioxidant and antiradical activity in the case of these compounds.

It cannot be considered that the 2,4-diphenolic fragment is inert and does not contribute to the activity of the molecules, but that it intervenes in a lesser extent than does the hydrazine function or other substituents present on the aromatic nucleus from position 4 of the thiazole heterocycle. This can be explained by two arguments: the OH groups are not adjacent (the catechol has a much higher antioxidant activity than non-adjacent ones) and the benzene ring is found near the electron withdrawing azomethine group, reducing the antioxidant potential of the phenols.

As described above, because the HOMO distribution was not similar in the case of the synthesized compounds, this interpretation was made according to this distribution. The highest HOMO-LUMO gap values were identified for compounds **5a** and **5b** (4.68 and 4.49 eV, respectively). Then, in the case of compounds having an aromatic nucleus in position 4 of the thiazole nucleus, this value decreased, starting from 4.46 eV (**6a**), 4.30 eV (**6b**), 4.19 eV (**7a** and **7b**), 4.12 eV (**8a**), and 3.92 eV (**8b**). We can see that the catechol substitution of the aromatic ring at position 4 of the thiazole is able to improve the activity of these compounds and the substitution of the azomethine carbon atom with hydrogen is similar to that of methyl.

**Figure 5:**
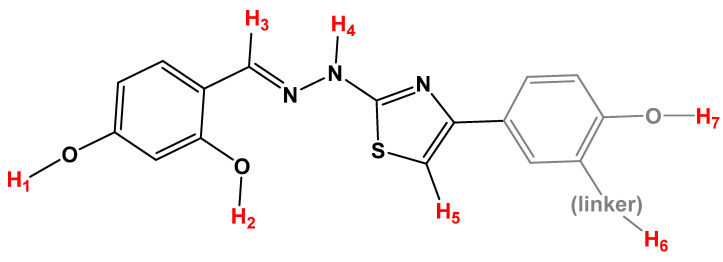
General structure of the compounds and the possible sites which can release hydrogen atoms to neutralize radicals numbered H_1_–H_7_.

In order to evaluate how the hydrogen can be released from the present molecules, the bond dissociation enthalpy (BDE) of O-H, N-H, and C-H bonds was computed from the possible molecular sites to identify which is the most susceptible hydrogen atom to be released. Obviously, the bond dissociation energies of hydrogen atoms bound to methyl groups or benzene rings were not calculated. Molecular sites likely to release hydrogen atoms were noted from H_1_ to H_7_, according to the general structure depicted in Figure 5. The computed X-H BDE (where X = N/C/O) from the H_1_–H_7_ available sites in the studied compounds are presented in Table 9. 

The most available site for releasing hydrogen atoms from our compounds is the hydrazine unit, numbered with H_4_ in the general structure, and the BDE computed for this site ranging between 62.3 kcal/mol (**8b**) to 67.3 kcal/mol (**5a**, **6a**). The second site that can release hydrogen atoms for the manifestation of the antiradical effect is represented by H_2_, which has slightly higher BDE values than H_4_, ranging between 65.2 kcal/mol (**8b**) and 70.2 kcal/mol (**5a**, **6a**). Analyzing in terms of value and order of BDE for H_2_ and H_4_, it can be seen that the two energies follow the same trend in the present series of compounds.

The analysis of the most stable conformations or the radical species at the H_4_ site resulted from quantum calculations indicated the formation of a hydrogen bond between the phenolic hydroxyl group from the *ortho* (H_2_) position and the azomethine nitrogen atom. This represents a form of intramolecular stabilization with the formation of a pseudo-bicyclic system, with high stability that favors extended conjugation, the stability of the resulting radical, and a better antiradical activity of the parent compound. Therefore, the presence of the OH group in the *ortho* position (H_2_ site) has a dual role: an antiradical role, known for phenolic compounds, but also stabilizing the hydrazone group to promote its antiradical activity and stabilize the resulting radical species. This observation is in agreement with previous literature reports [46,47].

The lowest BDEs are found in the **b** series of compounds, compared to analogous compounds in the **a** series; therefore, we consider that the presence of the methyl residue on the methylene carbon atom is unfavorable to the antiradical action. Because the H_1_ and H_4_ sites are spatially close to each other, the presence of the methyl residue can interfere with the internal stabilization of the radical resulting from the loss of the hydrogen atom from one of the hydrazine (H_4_) sites, probably by making the C=N double bond mor rigid, making it more difficult to bring the OH group in the *ortho* (H_2_ site) closer to the hydrazone nitrogen atom to form the intramolecular hydrogen bond to stabilize the resulting radical. 

Interesting, the H_1_ site corresponding to the phenolic OH group from *para* of both benzene rings is not considered to have significant potential to release hydrogen atoms, having slightly higher BDE values (75.8–81.4 kcal/mol). Although it does not act as a direct antiradical by releasing hydrogen atoms, it probably helps to stabilize the radicals resulting from the radicalization of H_2_ and H_4_ functions by its electron donor effect. 

In the case of the catechol group from compounds **8a-b**, the phenolic OH group from *meta* (H_6_) has a BDE lower than that from the *para* position (H_7_), and the value is close to those from H_2_ and H_4_. Therefore, in the case of compounds **8a-b** it can be considered that they have multiple groups within the same molecule that manifest the action, resulting in a compound with a very high antiradical activity; these fall into the category of hybrid molecules.

From the antiradical point of view, the methylene H_3_ site (**b** series of compounds), the thiazole H_5_ site (all compounds of the present paper), and the amide H_6_ site (compounds 7**a**-**b**) can be considered to be inert in terms of hydrogen atoms release.

### 3.5. Antimicrobial Activity Evaluation

The antimicrobial activity of the final compounds, against seven aerobic microbial strains (*S. aureus* ATCC 25923, *S. pyogenes* ATCC 19615, *P. aeruginosa* ATCC 27853, *S. enterica* ISM 83/37, *S. typhimurium* ATCC 14028, *E. coli* ATCC 8739*, E. coli* ATCC 25922), and a fungal strain (*C. albicans* ATCC 102310), was determined using a modified microdilution technique.

The results of the antimicrobial activity evaluation of the compounds, in comparison to norfloxacin and chloramphenicol used as standard antibiotics and fluconazole used as reference antifungal, are depicted in Table 10. Values over 100 µg/mL are replaced with “-“ and the compound was considered inactive on the respective microbial strain.

The synthesized compounds presented a modest antifungal activity, and compound **6b** was the only one active against the strain of *C. albicans* used for testing. Regarding the antibacterial activity, it can be observed that against the Gram-positive *S. aureus* and *S. pyogenes* bacterial strains, only compound **7a** presented inhibitory properties. With the exception of compound **6b**, all the other compounds were active against the Gram-negative *P. aeruginosa* strain, compounds **7a** and **8a** having the best antibacterial properties, presenting lower or equal MIC values to that of norfloxacin, which was used as a reference. These two compounds (**7a** and **8a**) also presented good cupric ions chelating activity, which may suggest a potential mechanism of antibacterial action, by subtracting transitional metal ions essential for bacterial metabolism. However, this needs further investigation.

### 3.6. Cytotoxicity Studies

#### 3.6.1. Cytotoxicity Determination

The IC_50_ values for the compounds against HaCaT cell lines were determined using the MTT viability test (Table 11). According to the IC_50_ values determined, the ranking of the compounds is as follows: **6b** > **8b** > **7a** > **5a** > **7b** > **8a** > **5b** > **6a**, where **6b** is the most cytotoxic compound (IC_50_ < 0.50 µg/mL) and **6a** is the least cytotoxic (IC_50_ = 126.76 µg/mL).

#### 3.6.2. HaCaT Cells’ Integrity

**Figure 6:**
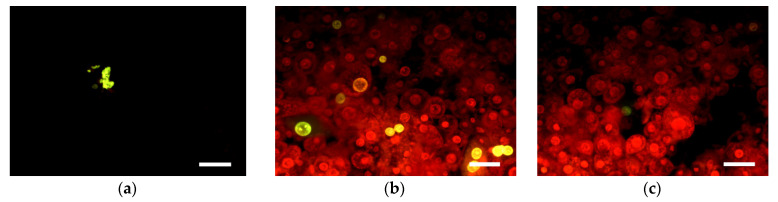

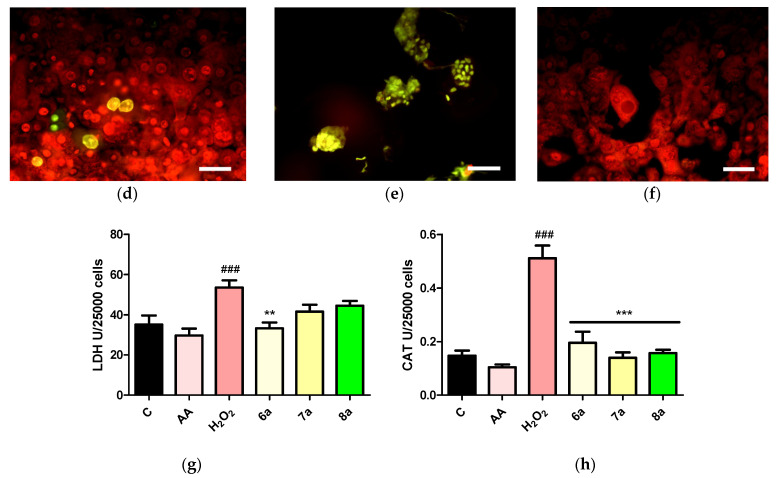
Acridine-orange staining (**a**–**f**) and enzymes assay (g-h) of the control and treated HaCaT cells: (**a**) control; (**b**) ascorbic acid (AA); (**c**) hydrogen peroxide (H_2_O_2_); (**d**) **6a**; (**e**) **8a**; (**f**) **7a**. Scale bar = 20 µm, × 400. Enzymes assays are presented for lactate dehydrogenase (LDH) (**g**) and catalase (CAT) (**h**) in U/no. of the cells. *p* < 0.05 was considered statistically significant. The significance was marked with ** for *p* < 0.01, *** for *p* < 0.001 (compared to H_2_O_2_ effects) and ^###^ for *p* < 0.001 for the comparison with the AA effects.

The acridine orange fluorescent signals of the cells were correlated with the IC_50_ values which described **7a** (Figure 6f) as most cytotoxic and **6a** (Figure 6d) as the least cytotoxic compound from the three selected compounds **6a**, **7a**, and **8a**. The red signals are specific for necroptotic cells when the dye is bound to acidic diffuse structures (e.g., lysed lysosomes) and suggest the cytotoxic microenvironment. In contrast, green fluorescence is for DNA and for viable nucleated cells. As compared to the AA (Figure 6b) and H_2_O_2_ (hydrogen peroxide) (Figure 6c) effects, **6a** action (Figure 6d) was similar to the AA effects when the nucleus was regular shaped, with green fluorescence without chromatin fragments. By contrast, the **8a** effects on HaCaT cells induced nuclear fragmentation (Figure 6e) and nucleus/cytoplasm disproportion, and suggested that the cells were orientated to necroptosis. The cellular action of the compound **7a** (Figure 6f) induced a necroptotic aspect of the cells, uniformized red signals, and acidic cytoplasm, which suggests cellular toxic events induced by **7a**. Interestingly, the LDH (Figure 6g) and CAT (Figure 6h) activities were decreased after the treatments with the tested compounds as compared to the H_2_O_2_ effects. The decreased activity of the LDH and CAT after the tested compounds treatment was explained as the bidirectional action of these compounds on the HaCaT cells. The compounds induced necroptotic status but, at the same time, the molecules protected the plasma membrane against oxidative stress attack and lysosome diffusion, as depicted in Figure 6d–f. In comparison to the AA action, these compounds acted in a slight cellular detrimental manner (increasing tendency for LDH and CAT, Figure 6g–h). Nevertheless, in oxidative conditions, the compounds act as antioxidants (**6a**) but with important cytotoxic effects (**7a**).

### 3.7. Molecular Properties with Influence on the Pharmacokinetics of Compounds

In order to obtain preliminary information about the pharmacokinetics of the compounds, some molecular parameters were computed and are presented in Table 12. Compounds **5a-b** and **6a-b** have a topological polar surface area (TPSA) equal to 105.98 Å**^2^**, below the limit of 140 Å**^2^** required to be considered as having a good absorption after oral administration. Compounds **8a-b**, slightly exceed the limit, having a TPSA value of 146.44 Å**^2^** and have an oral absorption of 58.48%, whereas compounds **7a-b** have the highest TPSA value in the present series, equal to 169.3 Å**^2^**, and a consecutive gastrointestinal absorption (GIA) equal to 50.59%, the smallest in this series of compounds.

Octanol-water partition coefficients were computed using multiple algorithms available in the Swiss ADME web tool. It can be seen (Table 12) that the compounds of series **a** have a higher lipophilicity than their structural analogues of series **b**, expressed by higher values of logP. Overall, the compounds did not violate any of Lipinski’s rules.

The pKa of the most acidic group of each compound is presented in Table 12. Although the studied compounds have several phenolic groups, the acidic character of these compounds (pKa ranging between 6.5 and 8.1) is lower than that of ascorbic acid (pKa = 4.2). This finding is important, especially linked with the results of the cytotoxicity study, because the acidic properties of the ascorbic acid can lead to damaging the cells, whereas in our compounds this effect is reduced due to the lower acidity of the chemical groups.

## 4. Conclusions

The in vitro antiradical and antioxidant evaluation revealed that all synthesized compounds presented significant activity. The most active compounds of these series were compounds **5a-b** and **8a-b**, and these findings are in accordance with the studied thermodynamic and electrochemical properties. The toxicity screening identified that the phenylethylidene derivatives (series **a**) were better tolerated than the benzylidene derivatives (series **b**). Among the most active compounds in terms of antioxidant potential, compound **8a** emerged, which also presented a good tolerability and a notable anti-*Pseudomonas aeruginosa* ATCC 27853 activity, similar to that of norfloxacin used as a reference antibiotic. Even though compound **7a** displayed the best antibacterial activity in our series, and good antioxidant and antiradical properties, unfortunately it exhibited high toxicity. Although the ferric ions chelation activity of the compounds was low, the cupric ions chelation activity was noteworthy for compounds **5a-b**, **7a**, and **8a**. 

## Figures and Tables

**Table 1:** Results of the ABTS˙^+^ scavenging assay.

Compound	% of ABTS˙^+^ Scavenged					IC_50_ (µg/mL)
	0.5 µg/mL	0.75 µg/mL	1 µg/mL	1.5 µg/mL	2.5 µg/mL	
**5a**	32.42 *	37.23 *	41.36 *	49.77 *	70.60 *	1.45 *
**5b**	35.55	41.10	48.40	66.50	98.09	1.00
**6a**	4.75	7.92	12.68	22.21	36.01	3.34
**6b**	11.67	17.45	19.83	28.56	42.53	2.97
**7a**	11.62	17.29	22.69	30.78	49.37	2.52
**7b**	25.42	30.15	36.50	49.20	69.53	1.60
**8a**	25.40	33.32	41.07	58.72	90.97	1.25
**8b**	40.09	44.44	50.79	66.66	96.82	0.91
**Ascorbic acid**	9.56	14.32	20.61	38.24	64.58	1.97

* Data were reported previously [3].

**Table 2:** Results of the DPPH**˙** scavenging assay.

Compound	% of DPPH˙ Scavenged					IC_50_ (µg/mL)
	1 µg/mL	1.5 µg/mL	2 µg/mL	3 µg/mL	5 µg/mL	
**5a**	13.11 *	20.68 *	28.87 *	48.22 *	79.92 *	3.20 *
**5b**	16.46	24.42	34.44	52.10	87.08	2.90
**6a**	5.90	9.07	12.87	19.88	38.13	6.56
**6b**	10.60	13.62	17.23	23.89	41.22	6.23
**7a**	20.01	22.10	26.75	32.43	49.27	5.19
**7b**	24.01	26.00	30.41	35.81	51.80	4.84
**8a**	25.05	31.43	36.02	47.13	68.90	3.26
**8b**	39.38	47.41	54.37	67.84	91.38	1.72
**Ascorbic acid**	19.39	29.10	38.27	53.42	80.28	2.89
**Trolox**	10.83	17.10	20.56	31.91	53.67	4.68

* Data were reported previously [3].

**Table 3:** Results of the NO**˙** scavenging assay.

Compound	% of NO˙ Scavenged
**5a**	36.26
**5b**	35.63
**6a**	20.58
**6b**	28.35
**7a**	21.44
**7b**	26.33
**8a**	32.72
**8b**	46.30
**Gentisic acid**	47.33
**Trolox**	32.12

**Table 4:** Results of the Ferric Reducing Antioxidant Potential (FRAP), Total Antioxidant Capacity (TAC), and Reducing Power (RP) assays, expressed as % activity of ascorbic acid and trolox.

Compound	% of Activity of Ascorbic Acid			% of Activity of Trolox		
	FRAP	TAC	RP	FRAP	TAC	RP
**5a**	108.32 *	120.58 *	74.29 *	257.96 *	191.31 *	187.87 *
**5b**	111.30	144.79	76.28	230.74	212.14	192.51
**6a**	44.79	49.90	23.03	97.65	73.11	58.11
**6b**	48.24	73.53	24.47	105.18	107.73	61.77
**7a**	72.14	95.10	43.73	157.28	139.34	110.36
**7b**	80.05	109.59	45.90	174.53	160.56	115.84
**8a**	86.34	136.68	76.08	173.34	200.25	192.01
**8b**	99.71	150.48	85.03	215.31	220.47	214.58

* Data were reported previously [3].

**Table 5:** Results of the ferrous ions chelation capacity evaluation.

Compound	Chelation Capacity (%)			
	50 µg/mL	100 µg/mL	150 µg/mL	200 µg/mL
**5a**	0.27 *	5.06 *	11.17	21.69
**5b**	1.18	8.95	18.42	31.33
**6a**	0.07	7.14	14.50	25.03
**6b**	0.07	4.84	13.39	22.27
**7a**	0.07	1.73	2.29	3.88
**7b**	0.07	3.44	9.13	16.28
**8a**	7.73	12.28	17.60	22.64
**8b**	10.74	18.15	25.06	33.38
**EDTA-Na_2_**	20.86	57.32	>95.00	>95.00

* Data were reported previously [3].

**Table 6:** Results of the cupric ions chelation capacity evaluation.

Compound	Chelation Capacity (%)				
	3.36 µg/mL	6.72 µg/mL	10.08 µg/mL	13.44 µg/mL	16.80 µg/mL
**5a**	10.74	14.51	20.10	28.62	33.67
**5b**	11.58	15.60	22.76	28.99	34.10
**6a**	6.33	10.30	13.65	15.90	18.67
**6b**	3.84	6.80	10.44	14.34	17.07
**7a**	8.76	16.70	26.63	34.35	38.88
**7b**	7.23	11.41	14.46	19.40	22.72
**8a**	11.13	17.02	25.14	34.16	38.91
**8b**	7.34	11.97	16.62	20.30	26.71
**EDTA-Na_2_**	12.36	19.61	29.97	39.18	45.86

**Table 7:** Comparative presentation of the variation of peak currents and oxidation/reduction potentials for the tested compounds.

Compound	E_ox_ (V)	I_ox_ (μA)	E_ox_ (V)	I_ox_ (μA)	E_ox_ (V)	I_ox_ (μA)	E_Red_ (V)	I_Red_ (μA)	E_Red_ (V)	I_Red_ (μA)	E_Red_ (V)	I_Red_ (μA)
**5a**	-	-	0.384	50.85	0.632	19.21	−0.075	−6.62	-	-	0.256	−2.17
**5b**	-	-	0.425	69.35	0.632	5.87	-	-	0.085	−0.68	0.286	−2.86
**6a**	0.298	10.73	0.418	22.48	0.621	15.03	-	-	0.181	−6.84	0.379	−1.19
**6b**	0.296	5.28	0.467	44.99	-	-	−0.08	−2.22	0.070	−1.49	0.401	−5.91
**7a**	0.269	14.75	0.374	2.06	0.561	2.69	-	-	0.205	−4.65	-	-
**7b**	0.267	13.22	0.412	15.78	0.543	6.385	−1.11	−0.119	−1.45	0.078	−2.49	0.201
**8a**	0.174	21.51	0.386	37.08	0.520	4.87	-	-	0.101	−4.79	0.335	−38.91
**8b**	0.166	2.19	0.508	40.99	-	-	-	-	-	-	0.325	−40.37

**Table 8:** Results of the quantum calculations.

Compound	HOMO (eV)	LUMO (eV)	HOMO-LUMO Gap (eV)
**5a**	−5.63	−0.95	4.68
**5b**	−5.69	−1.20	4.49
**6a**	−5.52	−1.06	4.46
**6b**	−5.58	−1.28	4.30
**7a**	−5.41	−1.22	4.19
**7b**	−5.47	−1.28	4.19
**8a**	−5.18	−1.06	4.12
**8b**	−5.22	−1.30	3.92

**Table 9:** The computed BDE for the hydrogen atoms from the studied compounds (kcal/mol).

Compound	H_1_	H_2_	H_3_	H_4_	H_5_	H_6_	H_7_
**5a**	81.1	**70.2**	N/A	**67.3**	116.8	N/A	N/A
**5b**	80.8	**65.9**	99.0	**63.3**	116.9	N/A	N/A
**6a**	81.3	**70.2**	N/A	**67.3**	115.1	N/A	N/A
**6b**	81.0	**65.8**	99.0	**63.1**	116.1	N/A	N/A
**7a**	81.4	**69.4**	N/A	**66.6**	114.4	103.5	79.2
**7b**	81.0	**66.1**	99.0	**63.4**	115.2	96.9	81.4
**8a**	81.2	**69.4**	N/A	**66.5**	114.0	**69.2**	75.8
**8b**	79.5	**65.2**	99.0	**62.3**	114.7	**69.3**	76.0

N/A: not applicable. The lowest BDE values are marked in bold.

**Table 10:** Minimum inhibitory concentrations (MIC (µg/mL)) of the synthesized compounds.

Compound	*S. aureus* ATCC 25923	*S. pyogenes* ATCC 19615	*P. aeruginosa* ATCC 27853	*S. enterica* ISM 83/37	*S. typhimurium* *ATCC 14028*	*E. coli* ATCC 8739	*E. coli* ATCC 25922	*C. albicans* ATCC 102310
**5a**	-	-	31.2	-	-	-	-	-
**5b**	-	-	31.2	-	-	-	-	-
**6a**	-	-	31.2	-	-	-	-	-
**6b**	-	-	-	-	-	-	-	62.5
**7a**	62.4	31.2	**3.9**	-	-	-	-	-
**7b**	-	-	15.6	-	-	-	-	-
**8a**	-	-	**7.8**	-	-	-	-	-
**8b**	-	-	31.2	-	-	-	-	-
**Norfloxacin**	0.48	<0.48	7.8	<0.48	<0.48	<0.48	7.8	NT
**Chloramphenicol**	7.8	3.9	0.48	3.9	<0.48	7.8	3.9	NT
**Fluconazole**	NT	NT	NT	NT	NT	NT	NT	31.2

NT: not tested. The best MIC values are marked in bold.

**Table 11:** The IC_50_ values of the compounds against HaCaT cells determined through the MTT method.

Compound	IC_50_ (µg/mL)	IC_50_ (nM)
**5a**	30.70	102.41
**5b**	50.34	176.17
**6a**	126.76	311.99
**6b**	<0.50	<1.27
**7a**	35.13	75.50
**7b**	42.36	93.86
**8a**	44.59	113.22
**8b**	8.54	22.48
**Ascorbic acid**	22.77	129.29

**Table 12:** Molecular parameters of the compounds without hydro acid (CwHA) of the final salefied compounds with strong influence on the preliminary steps of pharmacokinetics of compounds.

CwHA	MW	HBA	HBD	TPSA (Å^2^)	GIA (%)	iLOGP	XLOGP3	WLOGP	MLOGP	pKa
**5a**	263.32	4	3	105.98	72.44	2.19	2.81	2.51	0.91	8.0
**5b**	249.29	4	3	105.98	72.44	1.33	2.59	2.12	0.63	8.1
**6a**	325.38	4	3	105.98	72.44	2.37	4.08	3.87	1.96	7.9
**6b**	311.36	4	3	105.98	72.44	1.74	3.85	3.48	1.72	8.1
**7a**	384.41	6	5	169.30	50.59	1.81	3.15	2.67	0.67	6.5
**7b**	370.38	6	5	169.30	50.59	1.98	2.93	2.28	0.43	6.5
**8a**	357.38	6	5	146.44	58.48	2.08	3.37	3.28	0.88	8.0
**8b**	343.36	6	5	146.44	58.48	1.18	3.14	2.89	0.64	7.9
**AA**	176.12	6	4	107.22	72.01	−0.05	−1.64	−1.41	−2.60	4.2

CwHA: compounds without hydro acid, MW: molecular weight, HBA: hydrogen bond acceptors, HBD: hydrogen bond donors, TPSA: topological polar surface area, GIA: gastrointestinal absorption, iLOGP: implicit octanol-water partition coefficient [48], XLOGP3: computational octanol-water partition coefficient calculated according to Cheng et al. [49], WLOGP: log *P* coefficient calculated by Wildman and Crippen method [50], MLOGP: Moriguchi octanol-water partition coefficient [51].

**Figure A1:**
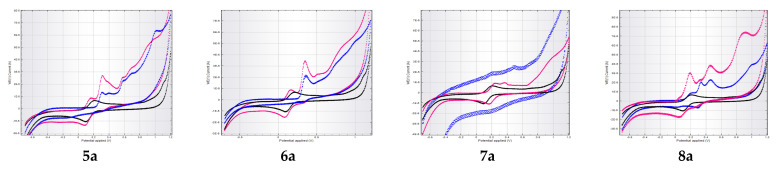

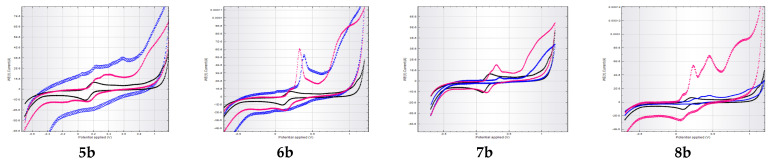
Overlapped CVs for 0.5 mM potassium ferricyanide (black); 0.5 mM solution of the novel synthesized compounds (blue) and of solutions with equal concentrations of 0.5 M of ferricyanide and one compound at a time (pink) prepared in 0.1 M HClO_4_ on graphite SPE.

**Figure A2:**
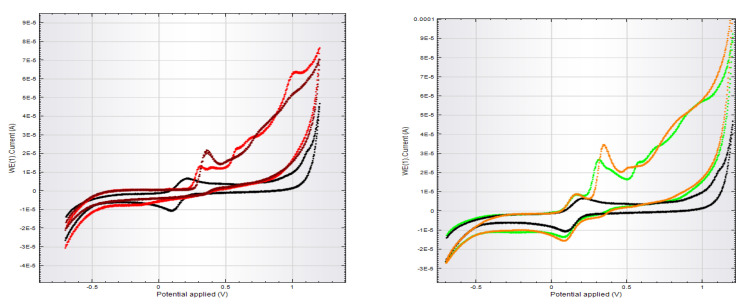
**Left**—Comparison between CVs for 0.5 mM of potassium ferricyanide (black), **5a** (red) and **5b** (brown). **Right**—Comparison between CVs for 0.5 mM of potassium ferricyanide (black); mixtures of 0.5 mM of potassium ferricyanide and 0.5 mM of **5a** (light green) and **5b** (orange).

**Figure A3:**
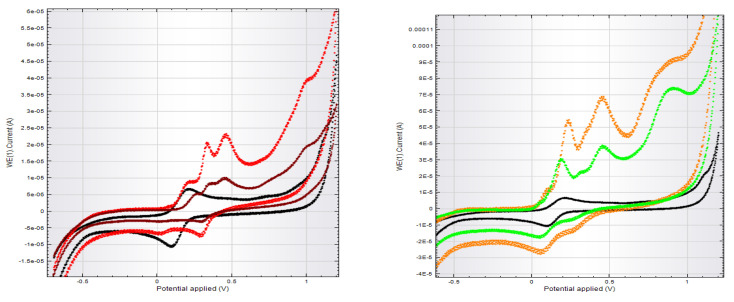
**Left**—Comparison between CVs for 0.5 mM of: potassium ferricyanide (black); **8a** (red) and **8b** (brown). **Right**—Comparison between CVs for 0.5 mM of potassium ferricyanide (black); and mixtures of 0.5 mM of potassium ferricyanide and: 0.5 mM of **8a** (light green) and **8b** (orange).

**Figure A4:**
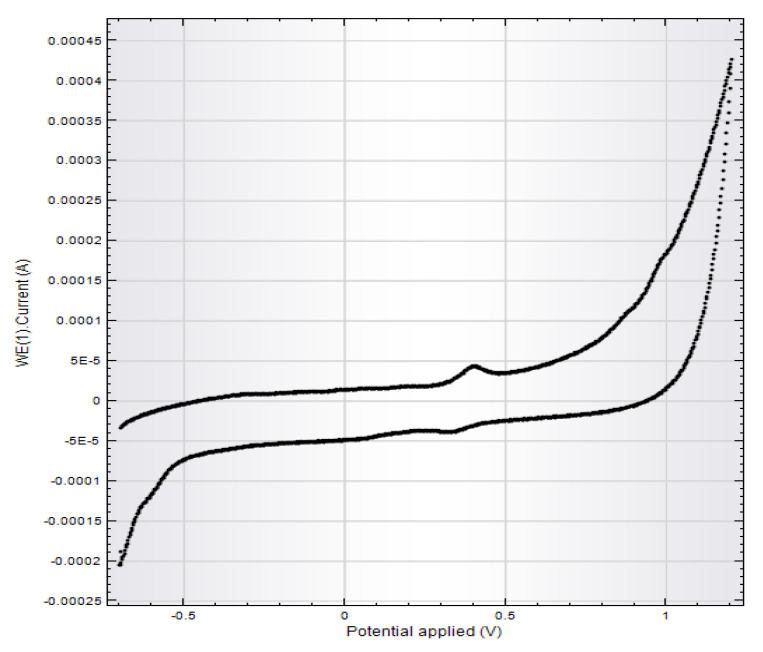
CV registered on graphite-based SPE in the presence of 0.5 mM DPPH˙ in 0.1 M HClO_4_.

**Figure A5:**
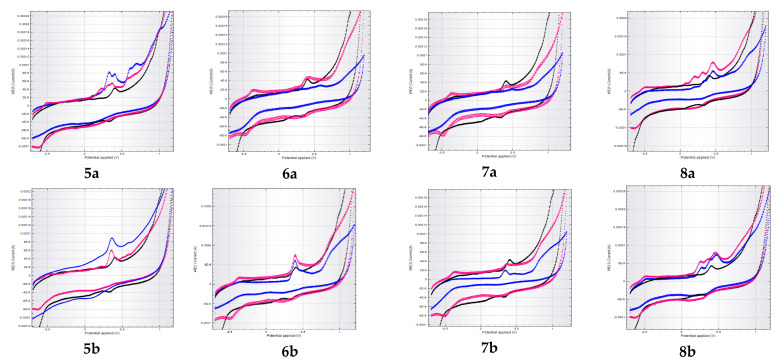
Overlapped CVs for 0.5 mM DPPH˙ (black); 0.5 mM solution of the synthesized compounds (blue) and of solutions with equal concentrations of 0.5 M of DPPH˙ and one compound at a time (pink) prepared in 0.1 M HClO_4_ on graphite SPE.

**Figure A6:**
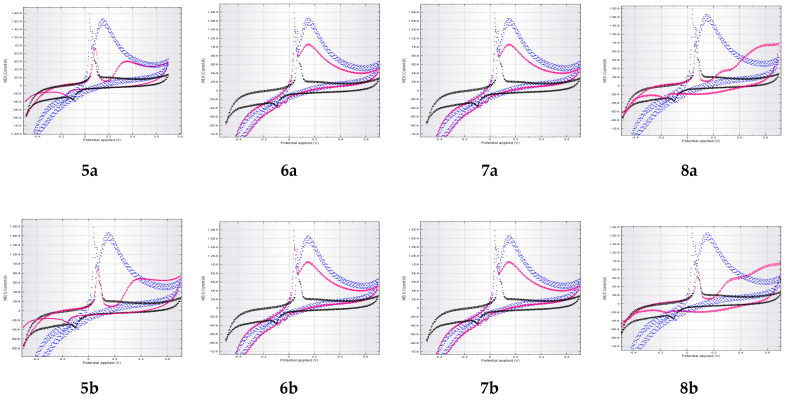
Overlapped CVs for the oxidation signal obtained for 0.5 mM H_2_O_2_ in the absence (blue) and in the presence 0.5 mM of the novel synthesized compounds (pink) prepared in 0.1 M KCl on Au SPE. Black line: CV recorded in 0.1 M KCl.

**Figure A7:**
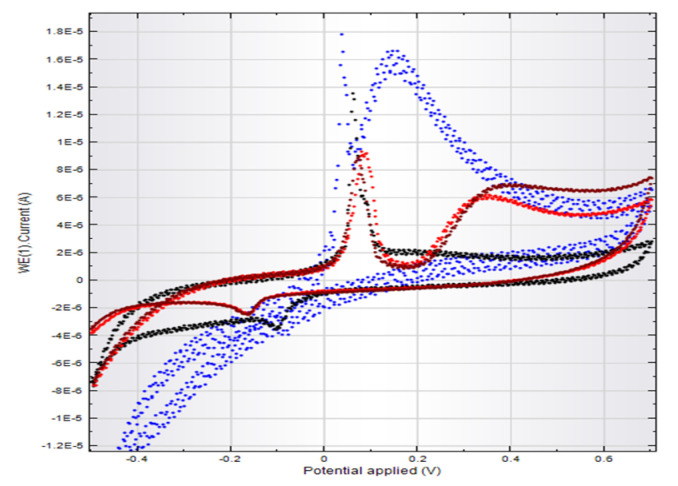
Overlapped CVs for the oxidation signal obtained for 0.5 mM H_2_O_2_ in the absence (blue) and in the presence of 0.5 mM of the novel synthesized compounds: **5a** (red) and **5b** (brown) prepared in 0.1 M KCl on Au SPE. Black line: CV recorded in 0.1 M KCl.

**Figure A8:**
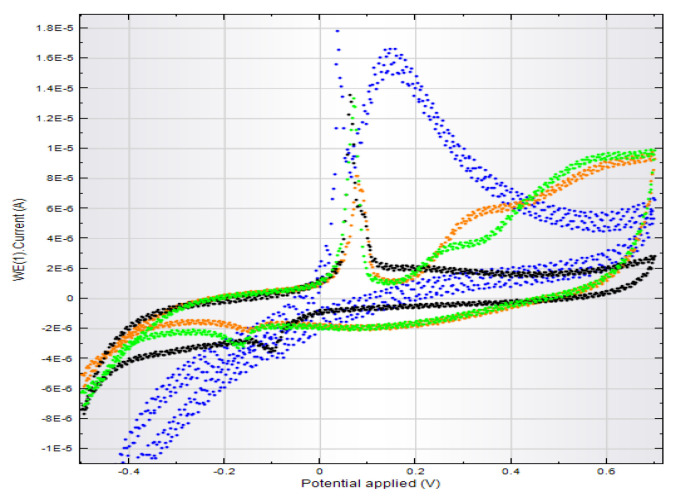
Overlapped CVs for the oxidation signal obtained for 0.5 mM H_2_O_2_ in the absence (blue) and in the presence of 0.5 mM of the novel synthesized compounds: **8a** (green) and **8b** (orange) prepared in 0.1 M KCl on Au SPE. Black line: CV recorded in 0.1 M KCl.

**Figure A9:**
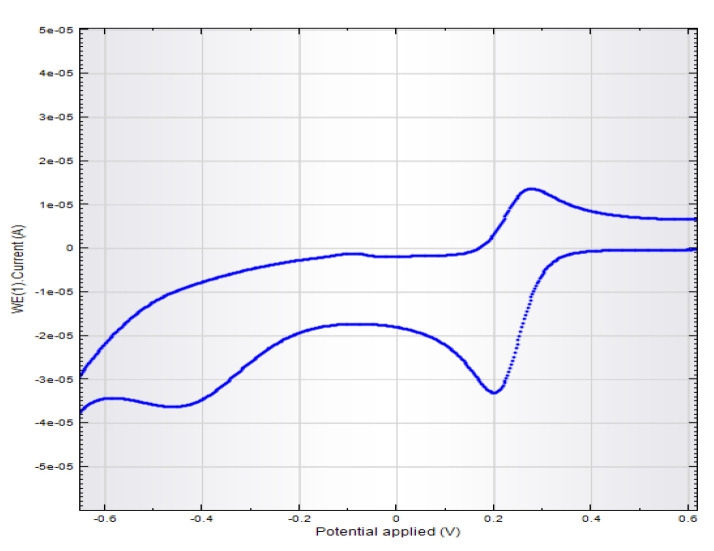
CV registered on graphite-based SPE in the presence of 2 mM TEMPO in 0.1 M HClO_4_.

**Figure A10:**
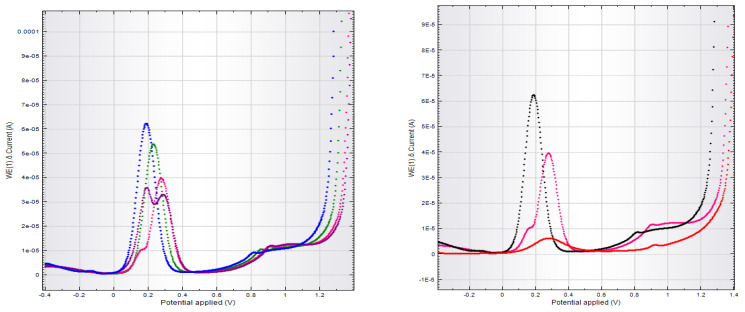
**Left**: DPV registered on graphite-based SPE in the presence of 2 mM TEMPO (blue) and for 2 mM TEMPO after one hour incubation with different concentrations of ascorbic acid: 0.25 mM (green); 0.5 mM (pink ) 1mM (purple). **Right**: DPVs registered on graphite-based SPE in the presence of 2 mM TEMPO (black); 0.5 mM ascorbic acid (red) and for 2 mM TEMPO after one hour incubation with 0.5 mM (pink) of ascorbic acid. Solutions are prepared in 0.1 M HClO_4_.

**Figure A11:**
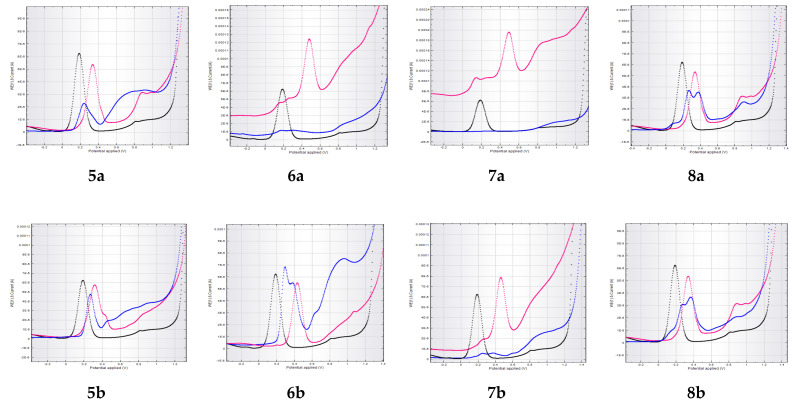
Overlapped DPVs for the oxidation signal obtained for 0.5 mM of the novel synthesized compounds (blue); 2 mM TEMPO (black) and after one hour incubation with 0.5 mM of the novel synthesized compounds (pink) on graphite-based SPE. Solutions were prepared in 0.1 M HClO_4_.

## Data Availability

Data supporting reported results can be found within the article, Appendix A or Supplementary Materials.

## Supplementary Materials

The following are available online at https://www.mdpi.com/article/10.3390/antiox10111707/s1, Table S1: HOMO and LUMO depicted for the compounds **5a-b, 6a-b, 7a-b** and **8a-b,** Figure S1: FTIR spectrum of compound **5b**, Figure S2: MS spectrum of compound **5b**, Figure S3: ^1^H-NMR spectrum of compound **5b**, Figure S4: ^13^C-NMR spectrum of compound **5b**, Figure S5: FTIR spectrum of compound **6a**, Figure S6: MS spectrum of compound **6a**, Figure S7: ^1^H-NMR spectrum of compound **6a**, Figure S8: ^13^C-NMR spectrum of compound **6a**, Figure S9. FTIR spectrum of compound **6b**, Figure S10: MS spectrum of compound **6b**, Figure S11: ^1^H-NMR spectrum of compound **6b**, Figure S12: ^13^C-NMR spectrum of compound **6b**, Figure S13: FTIR spectrum of compound **7a**, Figure S14: MS spectrum of compound **7a**, Figure S15: ^1^H-NMR spectrum of compound **7a**, Figure S16: ^13^C-NMR spectrum of compound **7a**, Figure S17: FTIR spectrum of compound **7b**, Figure S18: MS spectrum of compound **7b**, Figure S19: ^1^H-NMR spectrum of compound **7b**, Figure S20: ^13^C-NMR spectrum of compound **7b**, Figure S21: FTIR spectrum of compound **8a**, Figure S22: MS spectrum of compound **8a**, Figure S23: ^1^H-NMR spectrum of compound **8a**, Figure S24: ^13^C-NMR spectrum of compound **8a**, Figure S25: FTIR spectrum of compound **8b**, Figure S26: MS spectrum of compound **8b**, Figure S27: ^1^H-NMR spectrum of compound **8b**, Figure S28: ^13^C-NMR spectrum of compound **8b**.

## Appendix A

The electrochemical evaluation of the antioxidant capacity of compounds **5a-b**, **6a-b**, **7a-b**, and **8a-b** by comparison with control antioxidants is presented in Appendix A.

**Electrochemical evaluation of the antioxidant capacity of compounds 5a-b, 6a-b, 7a-b and 8a-b by comparison with control antioxidants**

1. **Ferric ions (Fe^3+^) reducing antioxidant power**

A common method used for in vitro determination of reducing capacity of compounds is Fe^3+^ reducing ability. The reducing power of a compound reflects the electron donating capacity and this may be associated with antioxidant activity.

This method is based on the evaluation of the influence of the compound measured by the direct reduction of Fe[(CN)_6_]^3−^ to Fe[(CN)_6_]^2−^. This assay is based on the reduction of Fe^3+^ to Fe^2+^ due to the action of antioxidants. Based on the electrochemical behavior of the studied compounds, the interaction of Fe^3+^ was followed by cyclic voltammetry (CV) via the possible changes of redox peaks characteristic for Fe^3+^/Fe^2+^ transition after the interaction with antioxidants (if any).

Figure A1 shows the voltammograms of 0.5 mM potassium ferricyanide in 0.1 M HClO_4_ (black), of each compound at 0.5 mM in 0.1 M HClO_4_ (blue) compared with that of the mixtures between potassium ferricyanide (0.5 mM) and of each component (0.5 mM) (pink). From the voltammogram of potassium ferricyanide it can be observed that the one-electron transfer process Fe^3+/^Fe^2+^ occurs at 0.247 V/ 0.150 V in acidic media (red) (Figure A1; Table A1).

Each compound presents its characteristic peaks (blue line in each figure). When the mixtures are evaluated (pink line in each figure), if a decrease of redox peaks typical for Fe^3+/^Fe^2+^ process is noticed, due to the interaction with one compound this suggests that that compound exhibits reducing action to ferricyanide ion and as a consequence antioxidant capacity.

Meantime, the oxidation signals of compounds can be influenced by the presence of ferricyanide in the solution, being partially oxidized during the interaction with Fe^3+^, and consequently, the electrochemical oxidation is slower as indicated by the shift of anodic peaks to higher potentials.

A different behavior can be observed again for the studied compounds, but again, **5a** has a similar behavior with **5b**, and **8a** with **8b**. These compounds were represented comparatively, individually, respectively in the presence of the redox probe (Figure A1, Figure A2 and Figure A3).

From Table A1 it can be seen the variation of the potential and intensity of the oxidation currents from one compound to another. In the case of **5a** and **5b**, it can be observed the decrease of the oxidation signal of the redox probe when the respective compounds are in the same solution, which is in accordance with the behavior of the compounds with antioxidant action, a phenomenon that occurs simultaneously with the increase of the oxidation signals corresponding to the compounds. In the case of **8a** and **8b** it is observed that both show an oxidation signal positioned in the same potential range as that of the redox probe. The interaction between **8a** or **8b** with the redox probe causes an almost complete decrease in the oxidation signal of the redox probe, confirming the antioxidant character of the compounds, while the oxidation signals of the compounds increase significantly.

It can be concluded that **8a** has the highest scavenging effect on ferric ion, followed by **8b**, **5b** and **5a** and that the variation of the oxidation signals of the tested compounds did not confirm the observed trend at the redox probe signal for all compounds tested. One explanation could be that almost all compounds have a signal in the potential range in which the ferric ion signal is, thus possible interference between them may occur.

**Table A1:** Comparative presentation of the scavenging effect of the synthesized compounds on ferricyanide.

Compound	E_ox_ (V)	I_ox_ (μA)	E_ox_ (V)	I_ox_ (μA)	E_ox_ (V)	I_ox_ (μA)	E_ox_ (V)	I_ox_ (μA)	E_ox_ (V)	I_ox_ (μA)	E_red_ (V)	I_red_ (μA)	E_red_ (V)	I_red_ (μA)
**Fe^3+^/Fe^2+^**	0.198	6.35	-	-	-	-	-	-	-	-	0.095	−7.98	-	-
**5a**	-	-	0.308	8.83	0.573	5.23			0.997	9.78			0.318	−1.23
**5a+Fe^3+^/Fe^2+^**	0.152	3.69	0.308	16.21	0.572	4.14			0.903	3.82	0.091	−7.33	0.323	−0.99
**5b**			0.355	15.68									0.327	−1.56
**5b+Fe^3+^/Fe^2+^**	0.152	5.57	0.342	22.3	0.505	1.20	0.506	8.68	0.806	8.68	0.093	−8.66	0.323	−1.52
**6a**	-	-	0.232	3.04	0.586	6.79								
**6a+Fe^3+^/Fe^2+^**			0.223	2.74	0.424	3.82					0.142	−5.88		
**6b**			0.386	37.55					0.894	8.86				
**6b+Fe^3+^/Fe^2+^**	0.203	2.48	0.325	44.78					0.879	21.84	0.149	−8.26		
**7a**			0.205	4.37	0.493	1.95								
**7a+Fe^3+^/Fe^2+^**			0.23	4.35	0.374	1.75					0.164	−7.23		
**7b**			0.347	2.07					1.065	2.64				
**7b+Fe^3+^/Fe^2+^**	0.205	1.39	0.305	6.08					0.926	6.34	0.161	−6.8		
**8a**			0.195	2.32	0.330	7.42	0.460	7.70	0.985	3.45			0.296	−5.09
**8a+Fe^3+^/Fe^2+^**	0.072	0.17	0.186	17.73			0.452	12.93	0.87	18.64	0.054	−8.37		
**8b**			0.249	1.02	0.349	1.76	0.450	2.29	0.989	1.17			0.303	−1.97
**8b+Fe^3+^/Fe^2+^**	0.083	0.34	0.227	28.09			0.449	28.48	0.79	15.19	0.079	−8.01	0.279	−2.81

2. **The DPPH˙ radical scavenging**

The radical scavenging properties of the novel compounds and the other antioxidants were determined using a stock DPPH˙ radical solution prepared in absolute ethanol. The antioxidant samples were mixed with DPPH˙ solution and HClO_4_ electrolyte and incubated in the dark for 30 min. The remaining redox activity of DPPH was measured by cycling the potential between −0.7 V and 1.2 V, with a scan rate of 0.1 V/s.

The nitrogen-centered 2-2-diphenyl-1-picrylhydrazyl radical (DPPH˙) based assay is one of the most commonly employed method to evaluate the ability of a compound to scavenge or neutralize free radicals based on the reduction of the radical. The electrochemical behavior of DPPH˙ was studied at graphite-based SPE in HClO_4_ (Figure A4) and the results showed that DPPH˙ is reduced and oxidized in a reversible manner as indicated by the redox peaks couple located at 0.396 V/0.333 V.

Taking into account the redox activity of DPPH˙, its diminution by an antioxidant can also be followed by CV, which is expected to show changes in the voltammetric peaks. Figure A5 reports the electrochemical study of initial electrooxidation of DPPH˙ and the novel compounds compared to remaining peaks after their incubation for 30 min. The decrease of all the peaks after mixture incubation indicates the partial consumption of DPPH˙.

As the charging current is much higher in the case of mixture, the changes of DPPH˙ peak are rather difficult to monitor.

In some situations the signals of DPPH˙ are very difficult to read due to the signals of the compounds, but it can be observed that the most important influence on it was again from the compounds **8b**, **5a**, **5b** and **8a**.

**Table A2:** Comparative presentation of the scavenging effect of the tested compounds and of some antioxidants on DPPH˙ radical after 30 min of reaction.

Compound	E_ox_ (V)	I_ox_ (μA)	E_ox_ (V)	I_ox_ (μA)	E_ox_ (V)	I_ox_ (μA)	E_ox_ (V)	I_ox_ (μA)	E_red_ (V)	I_red_ (μA)	E_red_ (V)	I_red_ (μA)
**DPPH**			0.396	16.9					0.333	−7.19		
**5a**			0.339	43.29	0.598	20.51	0.987	15.5			0.137	−9.59
**5a+DPPH**	0.218	8.68	0.362	13.23	0.572	11.81					0.188	−5.46
**5b**			0.357	31.76							0.11	−10.26
**5b+DPPH**			0.349	33.3							0.145	−7.56
**6a**	0.232	4.99			0.565	4.37					0.193	−4.16
**6a+DPPH**			0.379	18.21								
**6b**			0.391	47.3							0.239	−7.045
**6b+DPPH**			0.391	43.4					0.302	−1.09	0.425	−1.23
**7a**	0.205	3.21			0.493	3.95					0.217	−5.26
**7a+DPPH**			0.405	9.24								
**7b**			0.342	15.65							0.276	−2.54
**7b+DPPH**			0.359	9.076					0.291	−7.43		
**8a**	0.193	3.56	0.325	6.97	0.459	22.39	0.935	6.88			0.278	−18.04
**8a+DPPH**	0.188	11.53	0.311	11.18	0.455	20.68	0.908	6.39				
**8b**	0.259	10.92			0.47	29.61					0.127	−3.52
**8b+DPPH**	0.247	17.56			0.457	18.63			0.259	−14.96		

3. **Scavenging of hydrogen peroxide**

Hydrogen peroxide (H_2_O_2_) is not a free radical itself, but easily converts to free radicals like OH˙ in biological systems, where is then implicated as a highly damaging species in free radical pathology, capable of damaging almost every molecule of living cells.

The anodic oxidation of H_2_O_2_ was used to determine the antioxidant character of compounds in different samples. Gold-based SPEs purchased from Dropsens were used as working electrode for monitoring the change in the oxidation peak of hydrogen peroxide due to the addition of different amounts of the novel compounds. The oxidation signal of H_2_O_2_ obtained at the sweep of the potential in the range from −0.5 to 1.2 V on the gold electrode can be observed in Figure A6. The scavenging abilities of compounds from 1 to 8 on hydroxyl radical are compared in this figure. The addition of an antioxidant, by its ability to consume H_2_O_2_, determines the decrease of anodic peak characteristic for H_2_O_2_. It could be claimed that antioxidants act by reacting with the dissociated hydrogen peroxide and thus decreasing its concentration, producing a decrease in the anodic peak current that can be related to the antioxidant activity. However, this implies that the antioxidant must react with H_2_O_2_ in the solution.

Diverse hypotheses were proposed in literature in order to support the role of specific structural components as requisites for radical scavenging and antioxidant activity most of which refer to the configuration and total number of hydroxyl groups. It can be observed that **8a** has the highest scavenging effect on H_2_O_2_, followed by **8b, 5a** and **5b**.

4. **2,2,6,6-tetramethylpiperidinyl-1-oxy (TEMPO) scavenging**

TEMPO (2,2,6,6-tetramethylpiperidine-N-oxyl) is a stable organic nitroxide radical that has been reported to scavenge carbon-centered radicals. The CV of TEMPO in HClO_4_ (Figure A9) reveals its reversible one-electron transfer process with oxidation peak at 0.27 V corresponding to the oxidation of TEMPO to oxoammonium species, accompanied by a cathodic peak at 0.20 V corresponding to the reduction of electrochemically generated oxoammonium.

Differential pulse voltammetry (DPV) was then applied in order to evaluate the antioxidant effectiveness of the novel compounds through their ability to scavenge the free radical species. Figure A10-left shows the DPVs of 2 mM TEMPO and of 2 mM TEMPO incubated for one hour with different concentrations of ascorbic acid. It can be observed that the anodic peak of TEMPO decreases when increasing the concentration of ascorbic acid, used as control antioxidant.

Similar effect was followed in the case of the studied compounds (Figure A11).

The corresponding oxidation signal for TEMPO does not decrease after one hour of reaction with compounds **6a**, **7a**, **6b** and **7b**. These compounds only cause the signal shift to higher potential values. In contrast, compounds **5a**, **8a**, **5b** and **8b** cause both the signal shift to higher oxidation potentials and its decrease, this behavior being similar to that observed in the case of ascorbic acid, the control antioxidant.
