# Supplementary material for: Phenolic Thiazoles with Antioxidant and Antiradical Activity. Synthesis, In Vitro Evaluation, Toxicity, Electrochemical Behavior, Quantum Studies and Antimicrobial Screening
Source: Antioxidants (Basel). 2021 Oct 27;10(11):1707. doi: 10.3390/antiox10111707 (PMC8615111; doi:10.3390/antiox10111707)
Supplement: Supplementary file 1 [file antioxidants-10-01707-s001.zip › Supplementary Materials.pdf]

Supplementary Materials

Phenolic Thiazoles with Antioxidant and Antiradical Activity. Synthesis, In Vitro Evaluation, Toxicity, Electrochemical Behavior, Quantum Studies and Antimicrobial Screening

Gabriel Marc [1], Anca Stana [1,*], Ana Horiana Franchini [1], Dan Cristian Vodnar [2,*], Gabriel Barta [2], Mihaela Tertiş [3], Iulia Şanta [3], Cecilia Cristea [3], Adrian Pîrnău [4], Alexandra Ciorîţă [4,5], Bogdan Dume [5], Vlad-Alexandru Toma [4,5,6], Laurian Vlase [7], Ilioara Oniga [8] and Ovidiu Oniga [1]

1. Department of Pharmaceutical Chemistry, "Iuliu Haţieganu" University of Medicine and Pharmacy, 41 Victor Babeş Street, RO-400012 Cluj-Napoca, Romania; marc.gabriel@umfcluj.ro (G.M.); ana.ho.franchini@elearn.umfcluj.ro (A.H.F.); ooniga@umfcluj.ro (O.O.)
2. Department of Food Science and Technology, University of Agricultural Sciences and Veterinary Medicine, 3-5 Manăştur Street, RO-400372 Cluj-Napoca, Romania; gabriel.barta@usamvcluj.ro
3. Department of Analytical Chemistry, Faculty of Pharmacy, "Iuliu Haţieganu" University of Medicine and Pharmacy, 4 Louis Pasteur Street, RO-400349 Cluj-Napoca, Romania; mihaela.tertis@umfcluj.ro (M.T.); iulia.nico.santa@elearn.umfcluj.ro (I.Ş.); ccristea@umfcluj.ro (C.C.)
4. National Institute for Research and Development of Isotopic and Molecular Technologies, 67-103 Donath Street, RO-400293 Cluj-Napoca, Romania; apirnau@itim-cj.ro (A.P.); alexandra.ciorita@itim-cj.ro (A.C.); vlad.toma@ubbcluj.ro (V.-A.T.)
5. Department of Molecular Biology and Biotechnologies, Babeş-Bolyai University, 4-6 Clinicilor Street, RO-400006 Cluj-Napoca, Romania; bogdan.dume@stud.ubbcluj.ro
6. NIRDBS Bucharest, Institute of Biological Research, 48 Republicii Street, RO-400015 Cluj-Napoca, Romania
7. Department of Pharmaceutical Technology and Biopharmaceutics, "Iuliu Haţieganu" University of Medicine and Pharmacy, 41 Victor Babeş Street, RO-400012 Cluj-Napoca, Romania; laurian.vlase@umfcluj.ro
8. Department of Pharmacognosy, "Iuliu Haţieganu" University of Medicine and Pharmacy, 12 Ion Creangă Street, RO-400010 Cluj-Napoca, Romania; ioniga@umfcluj.ro

* Correspondence: stana.anca@umfcluj.ro (A.S.); dan.vodnar@usamvcluj.ro (D.C.V.); Tel.: +40-264-450-529 (A.S.); +40-747-341-881 (D.C.V.)

Theoretical Quantum Studies

Table S1. HOMO and LUMO depicted for the compounds **5a-b, 6a-b, 7a-b** and **8a-b**.

Compound	HOMO	LUMO
**5a**	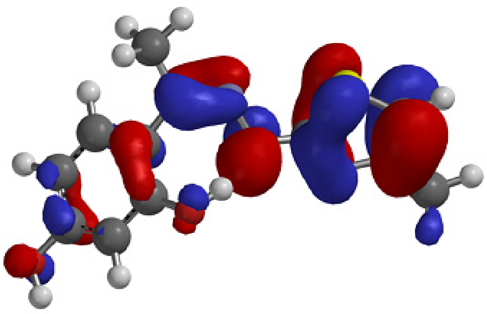	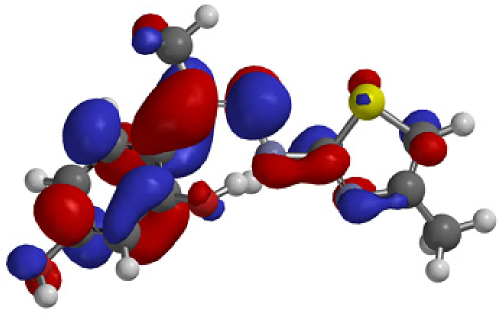
**5b**	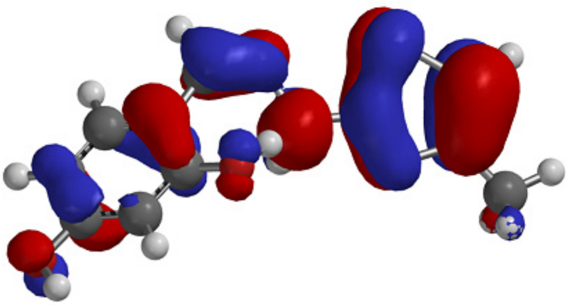	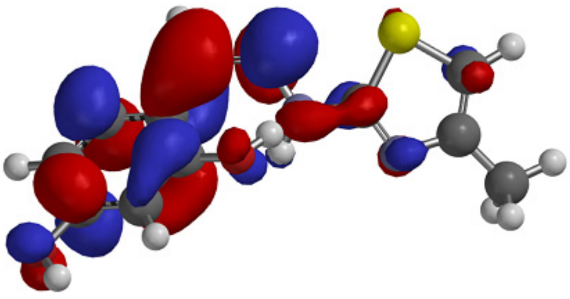

**6a**	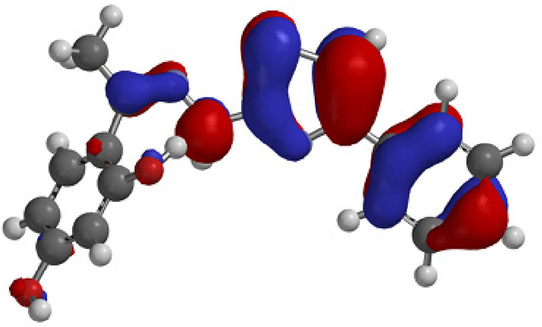	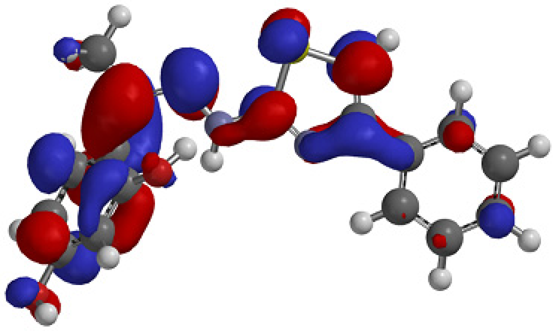
**6b**	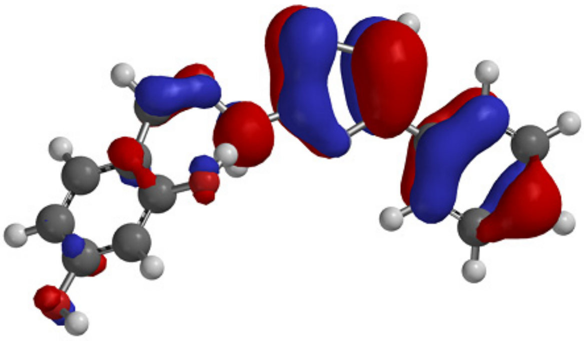	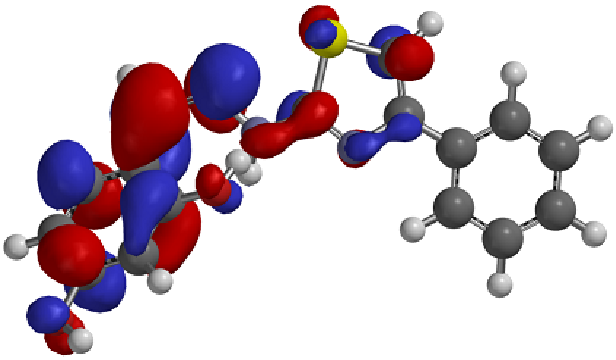
**7a**	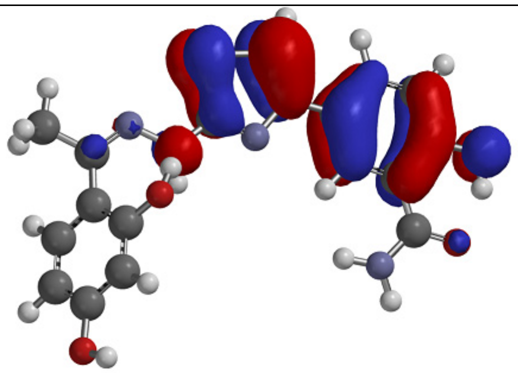	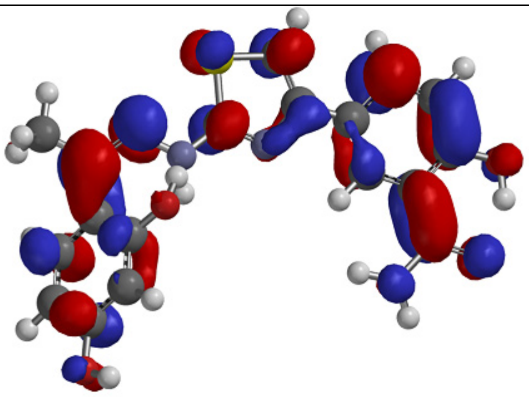
**7b**	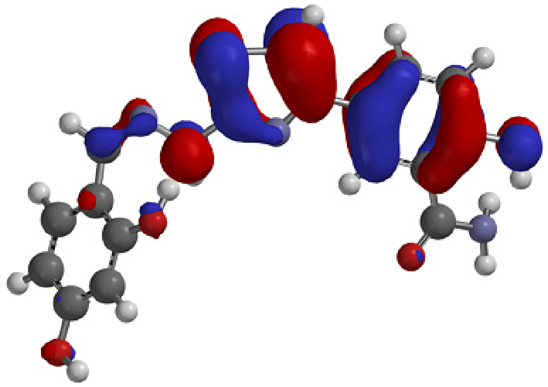	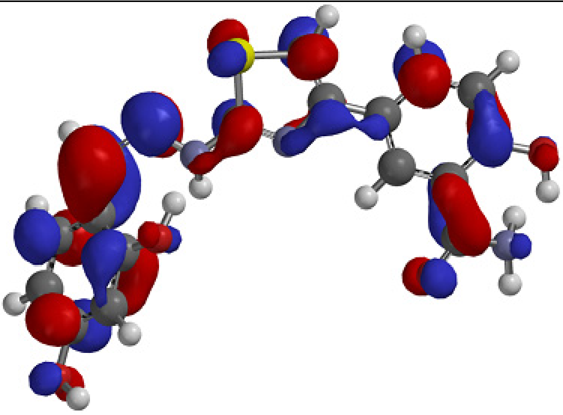

**8a**	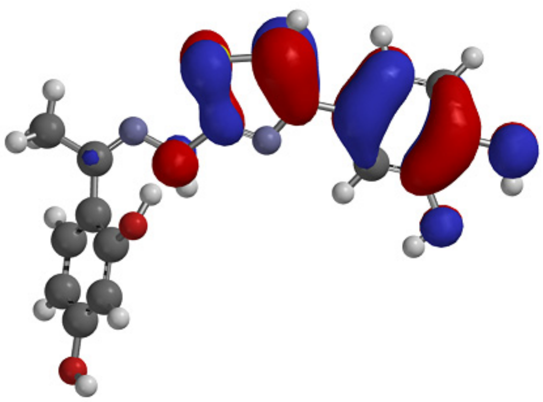	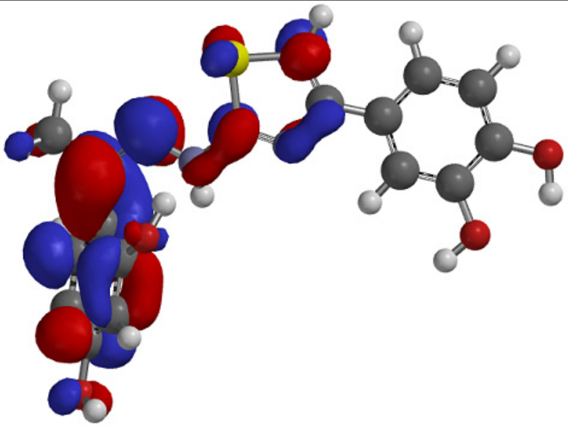
**8b**	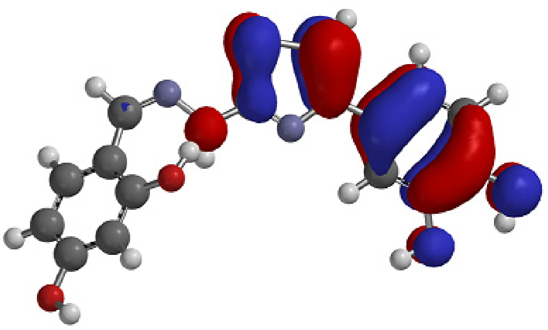	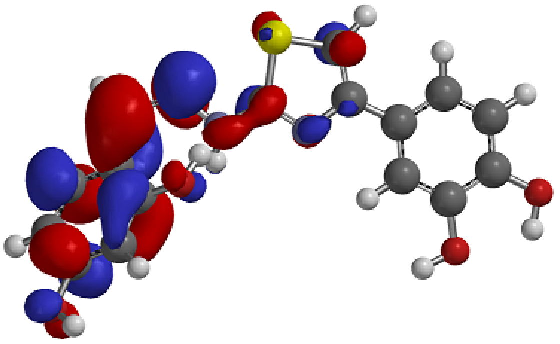

Spectral data for the compounds 5b, 6a-b, 7a-b and 8a-b

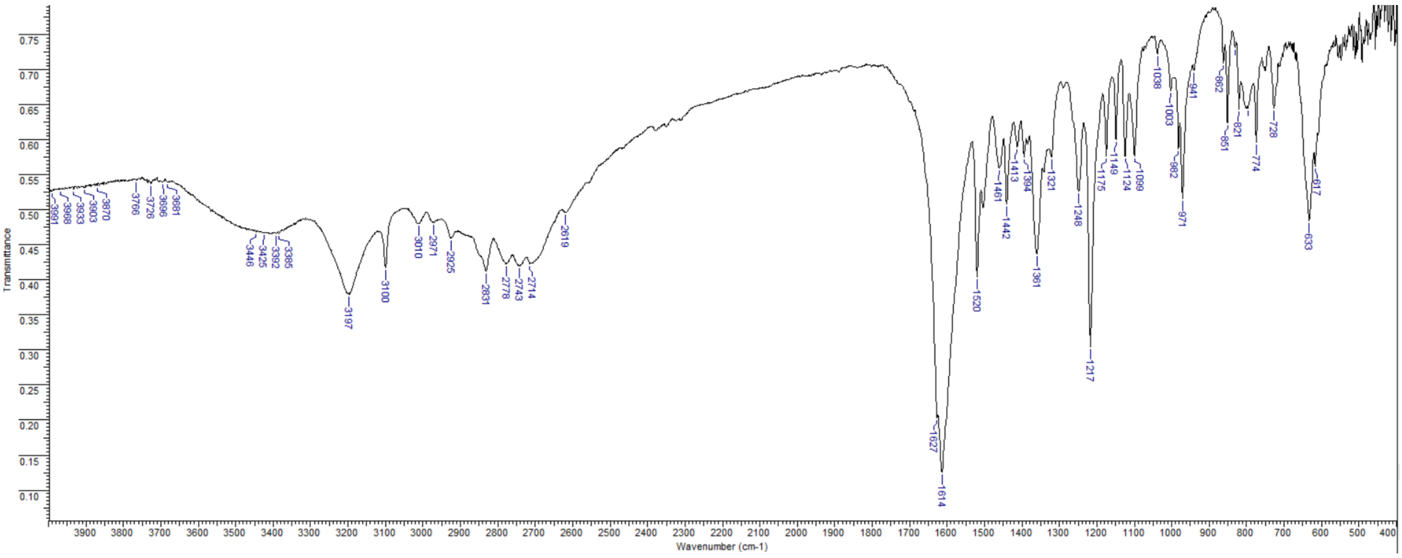

Figure S1. FTIR spectrum of compound **5b**.

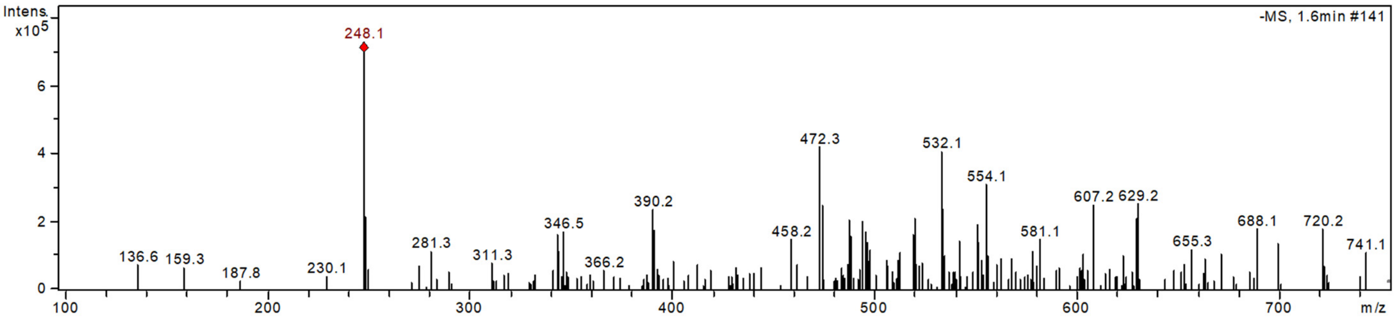

Figure S2. MS spectrum of compound **5b**.

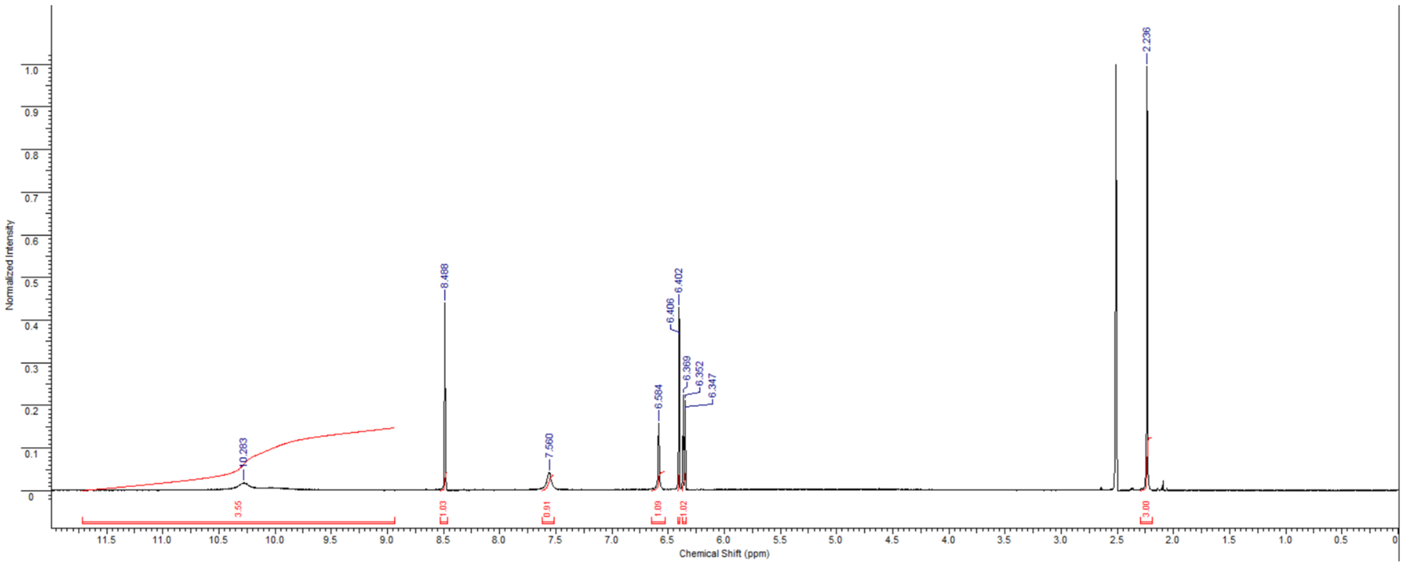

Figure S3. ^{1}H-NMR spectrum of compound **5b**.

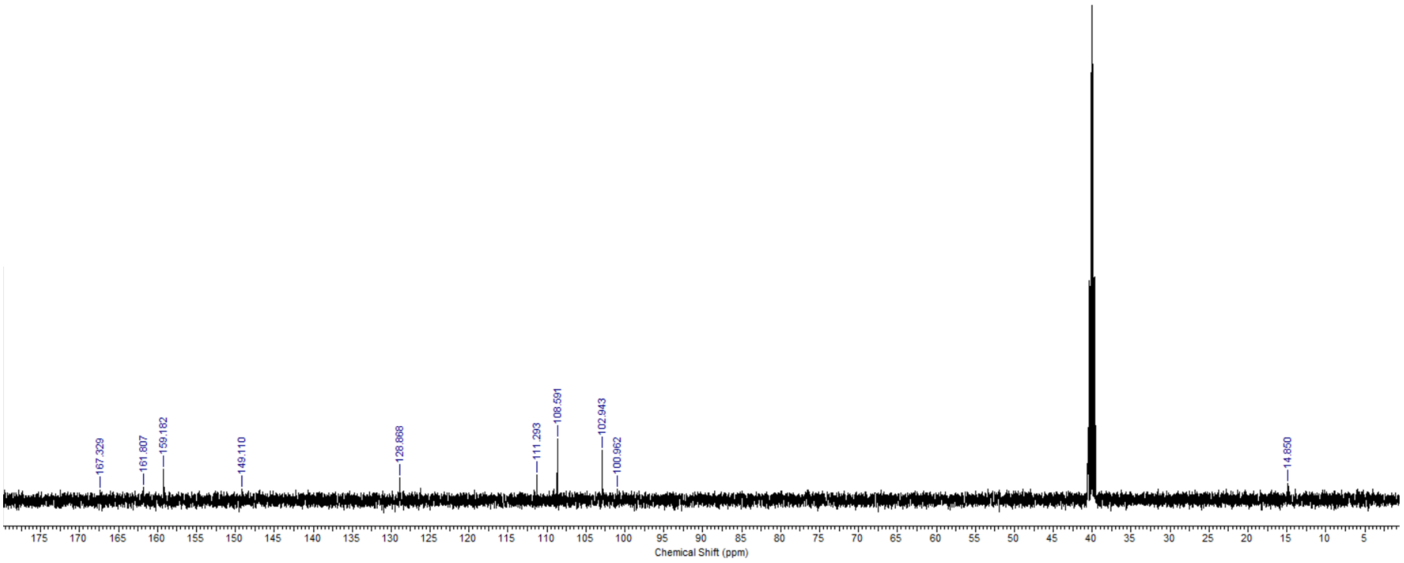

Figure S4. ^{13}C-NMR spectrum of compound **5b**.

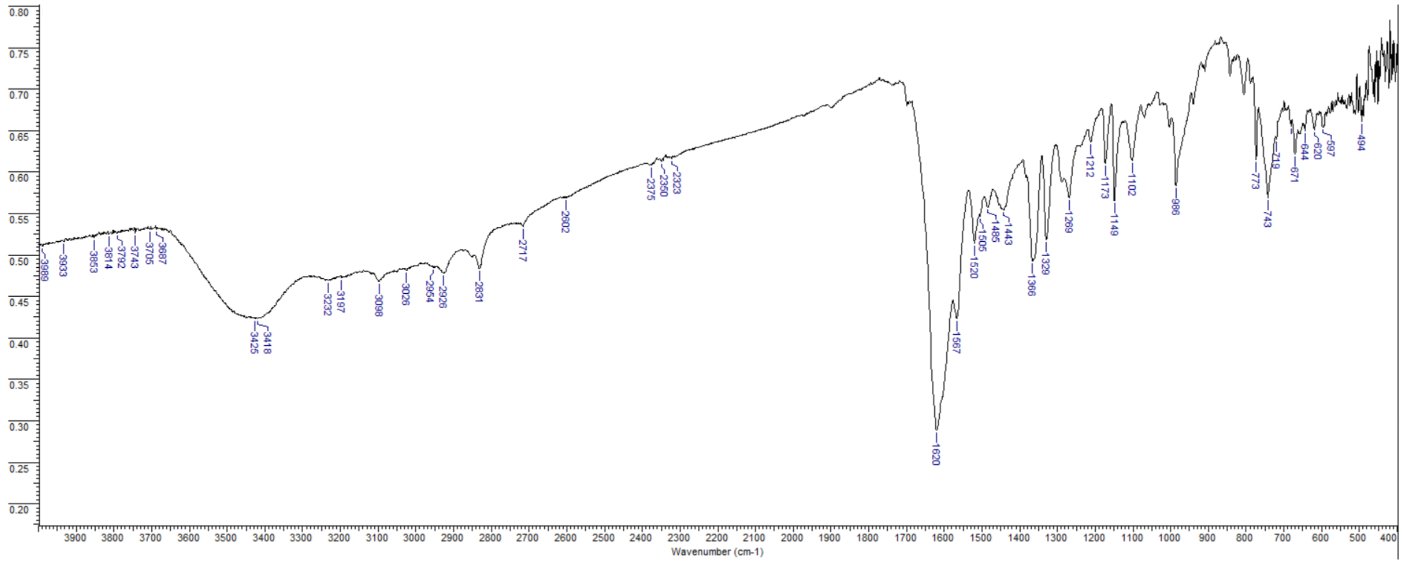

Figure S5. FTIR spectrum of compound **6a**.

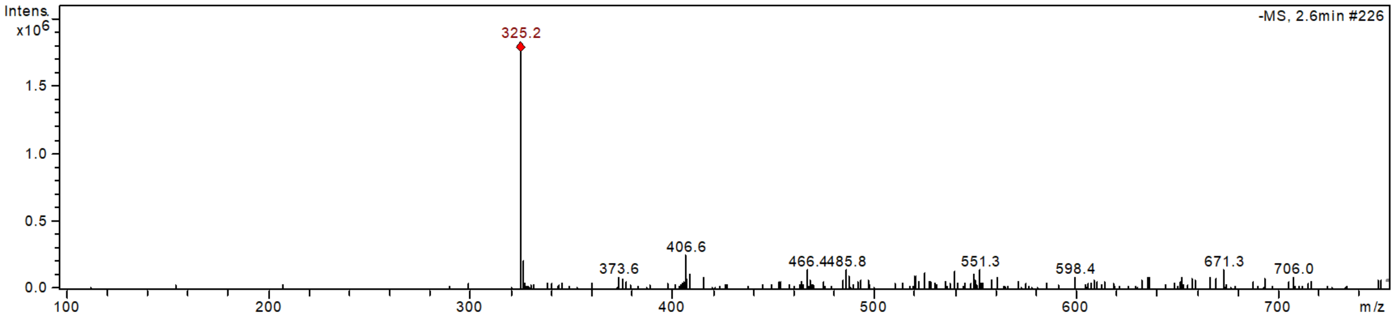

Figure S6. MS spectrum of compound **6a**.

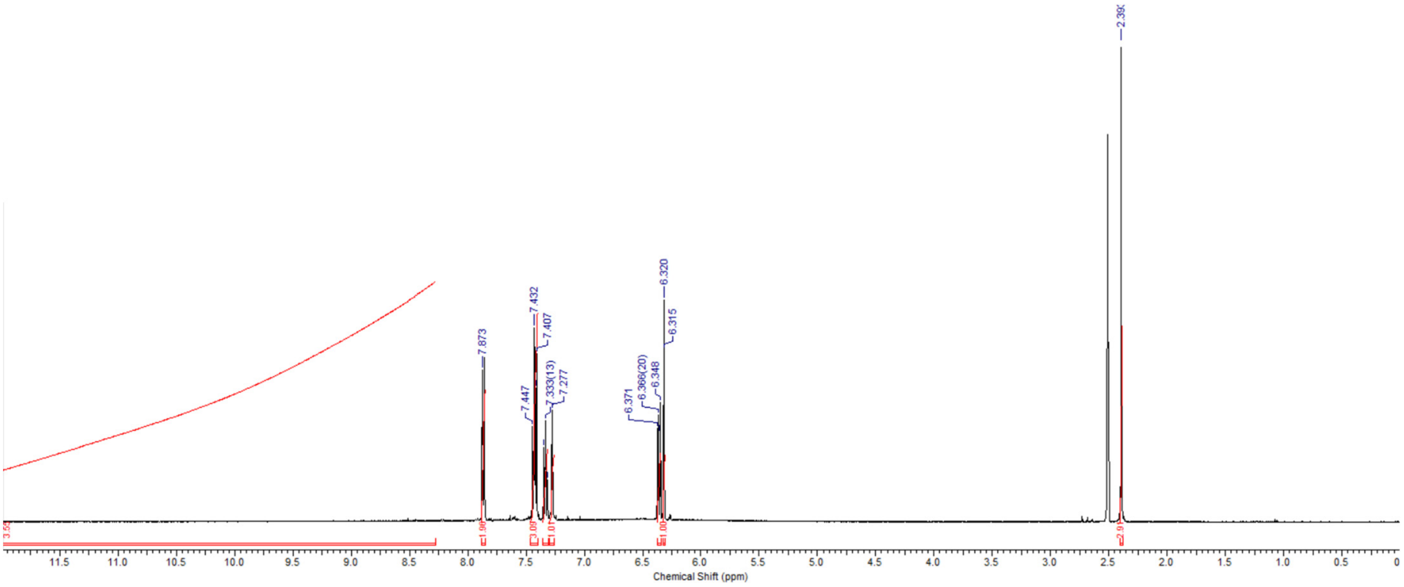

Figure S7. ^{1}H-NMR spectrum of compound **6a**.

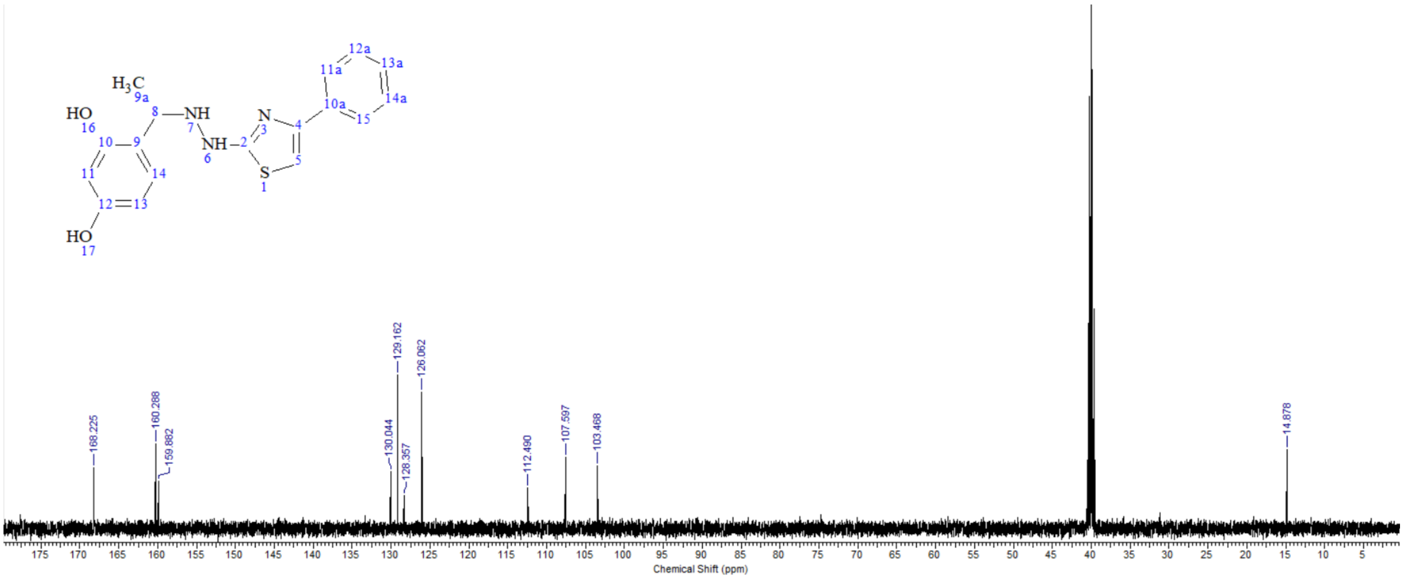

Figure S8. ^{13}C-NMR spectrum of compound **6a**.

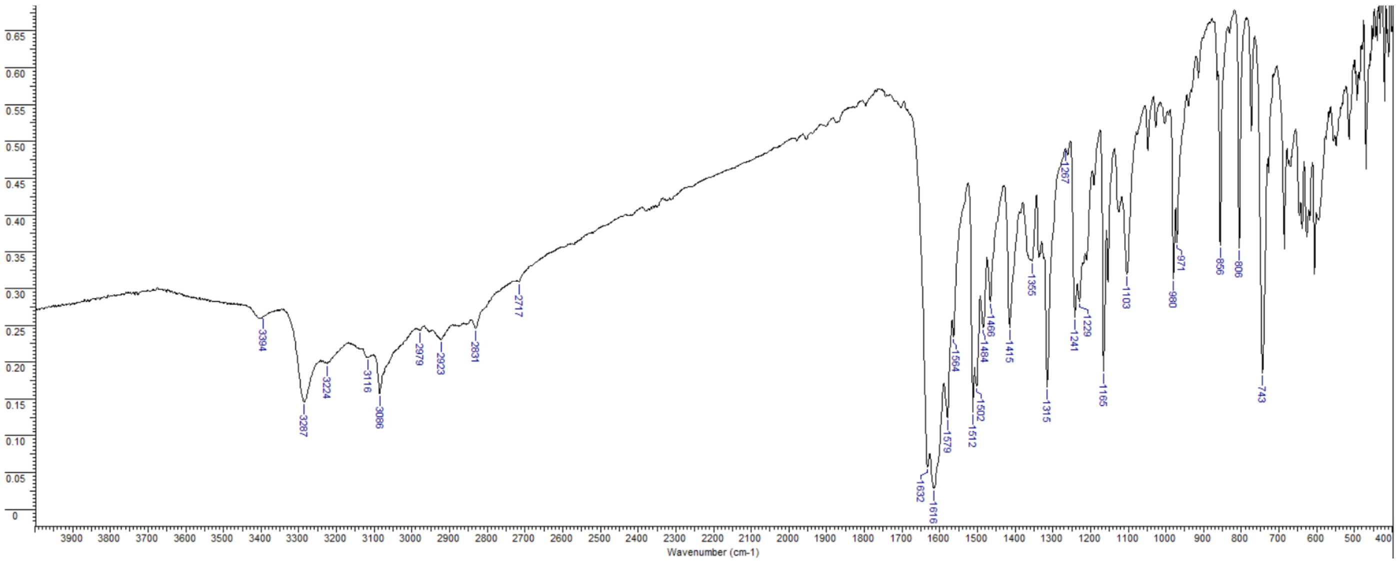

Figure S9. FTIR spectrum of compound **6b**.

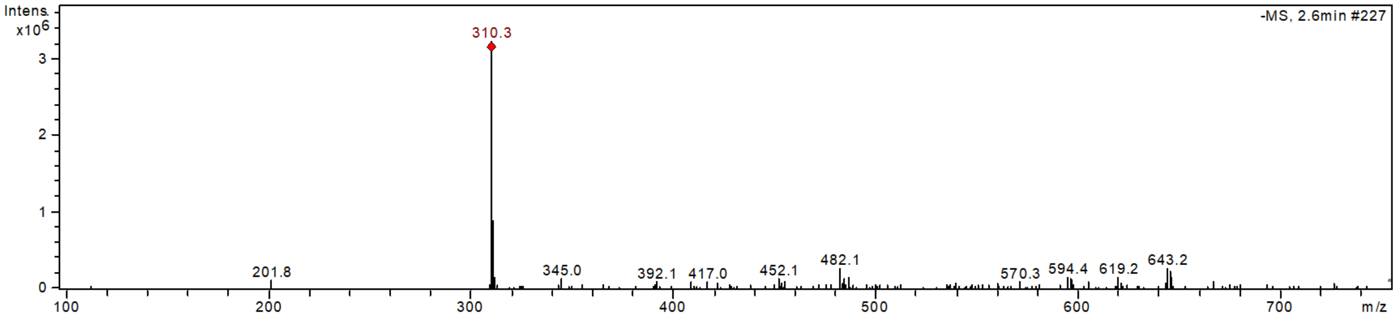

Figure S10. MS spectrum of compound **6b**.

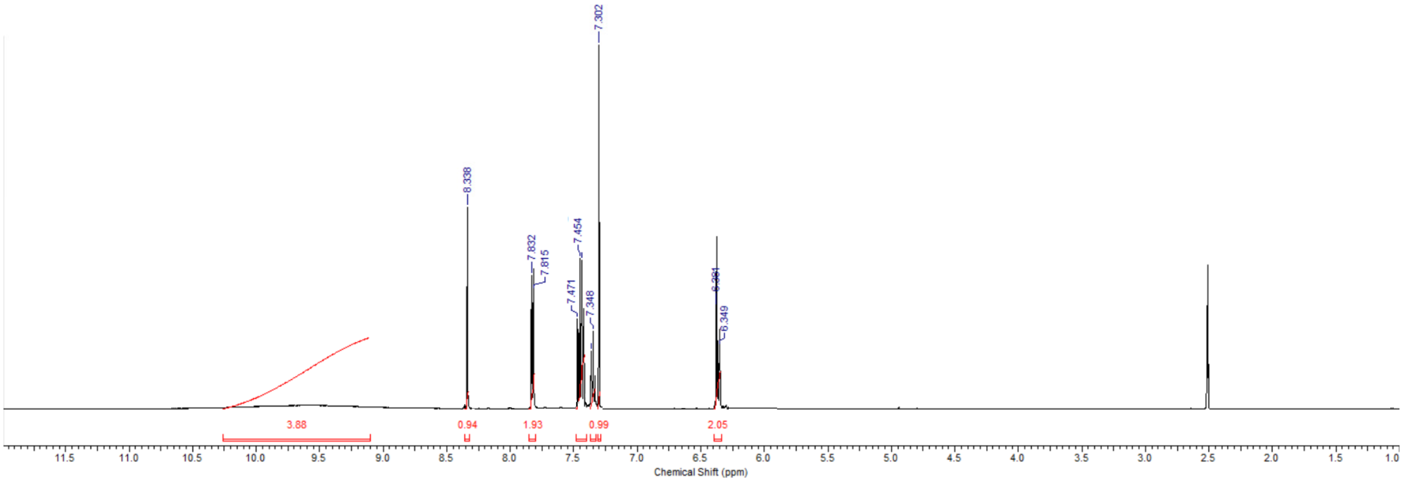

Figure S11. ^{1}H-NMR spectrum of compound **6b**.

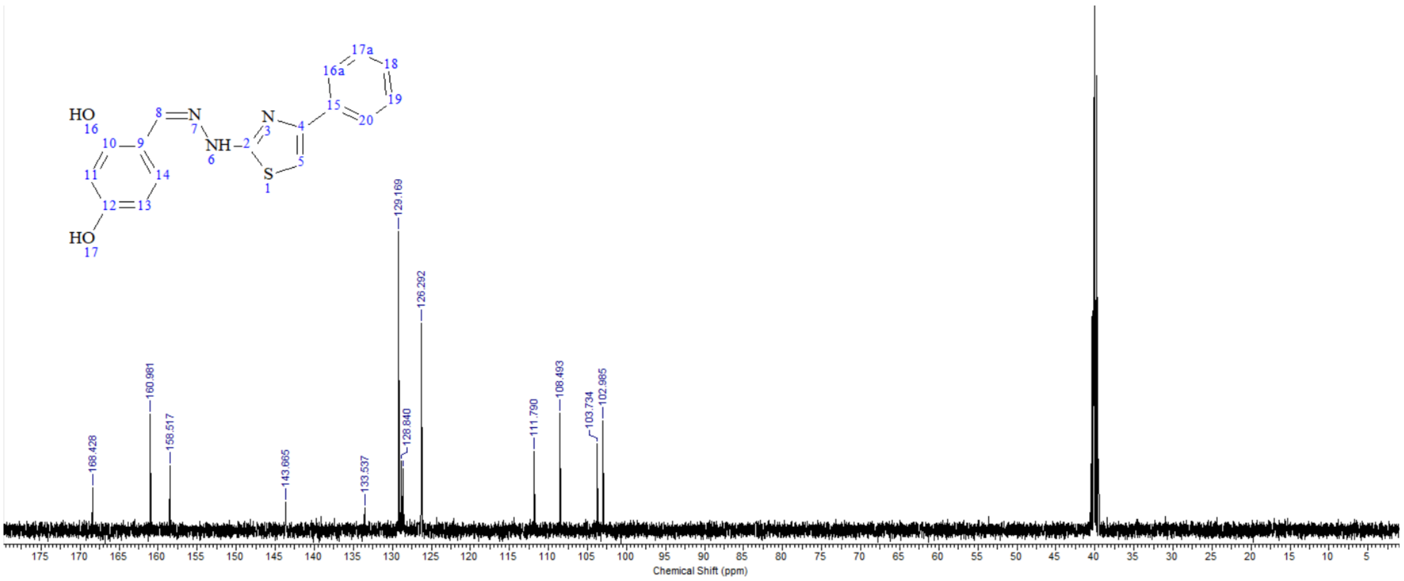

Figure S12. ^{13}C-NMR spectrum of compound **6b**.

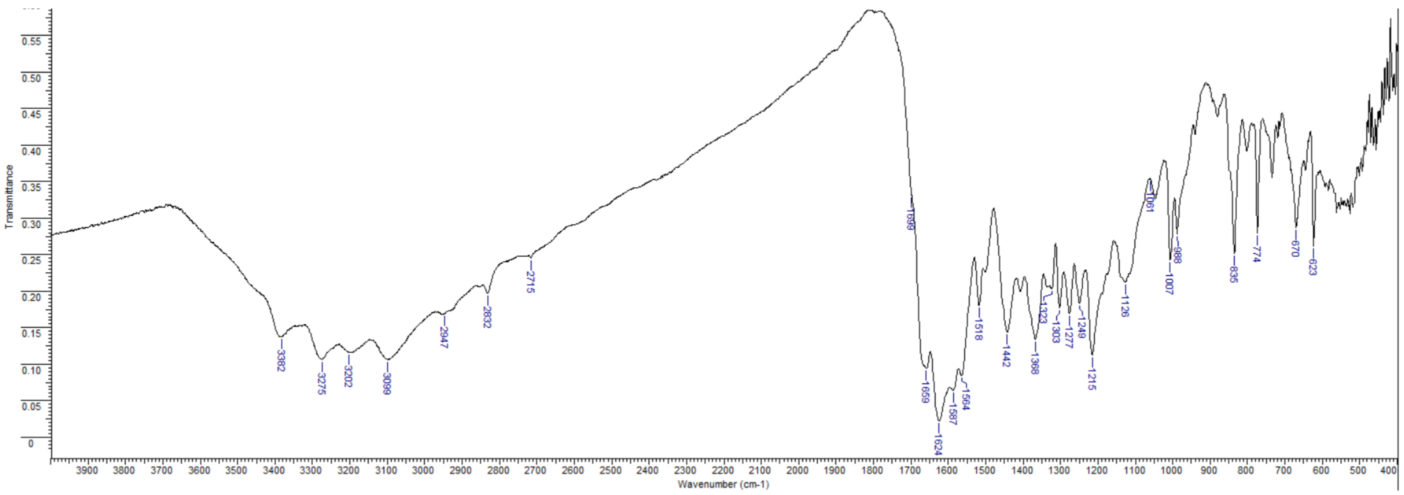

Figure S13. FTIR spectrum of compound **7a**.

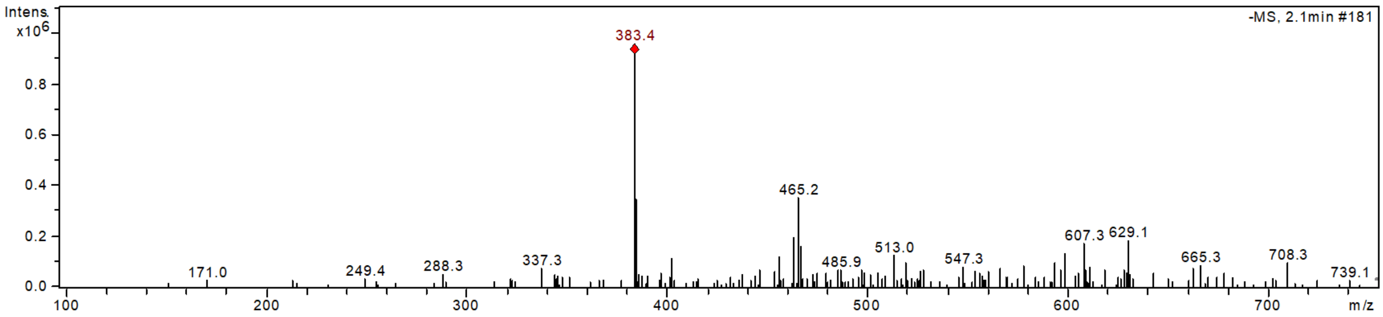

Figure S14. MS spectrum of compound **7a**.

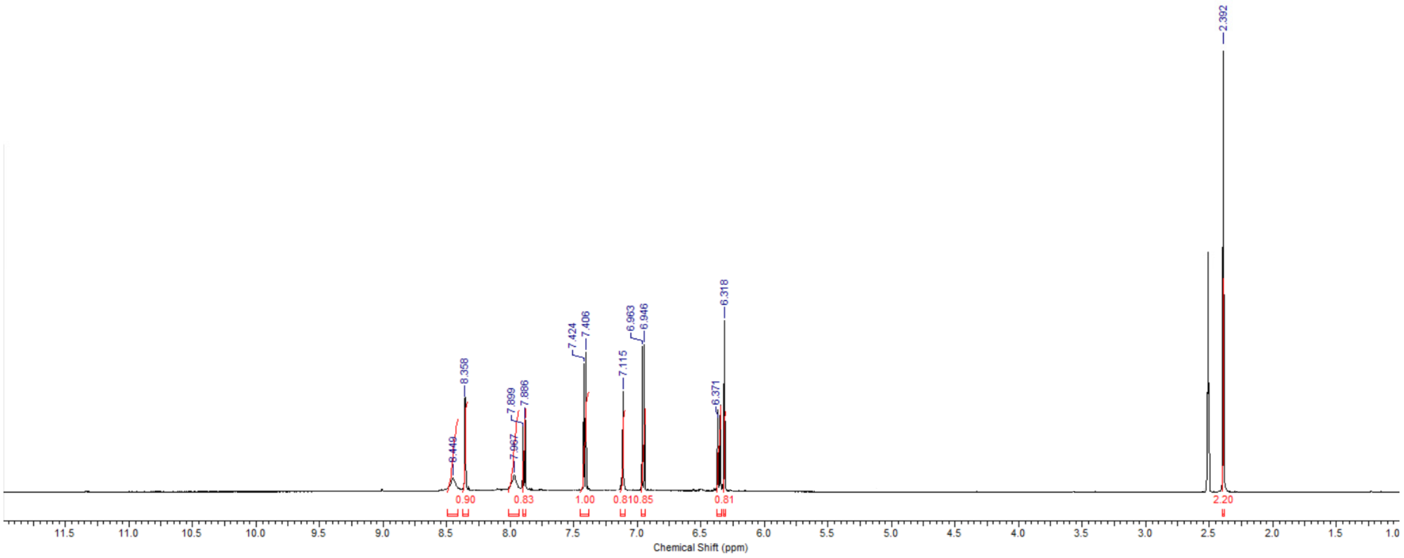

Figure S15. ^{1}H-NMR spectrum of compound **7a**.

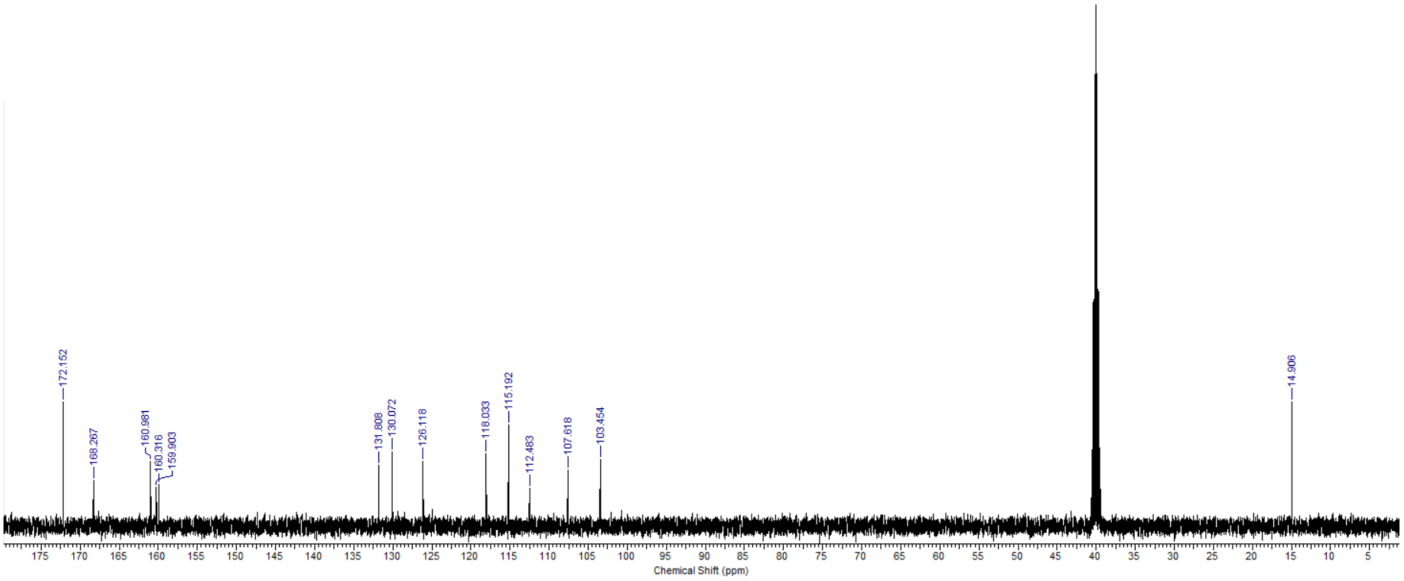

Figure S16. ^{13}C-NMR spectrum of compound **7a**.

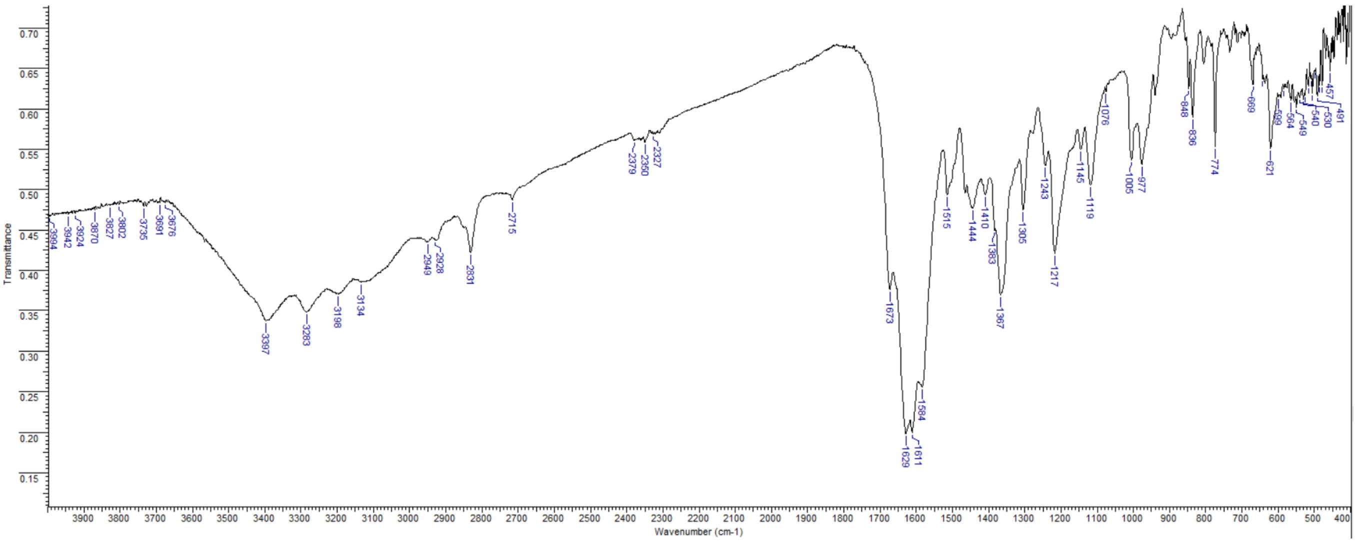

Figure S17. FTIR spectrum of compound **7b**.

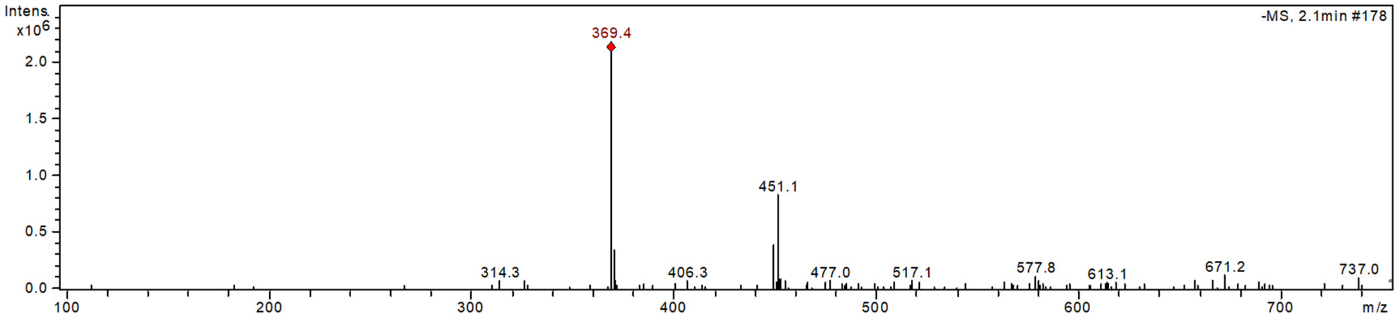

Figure S18. MS spectrum of compound **7b**.

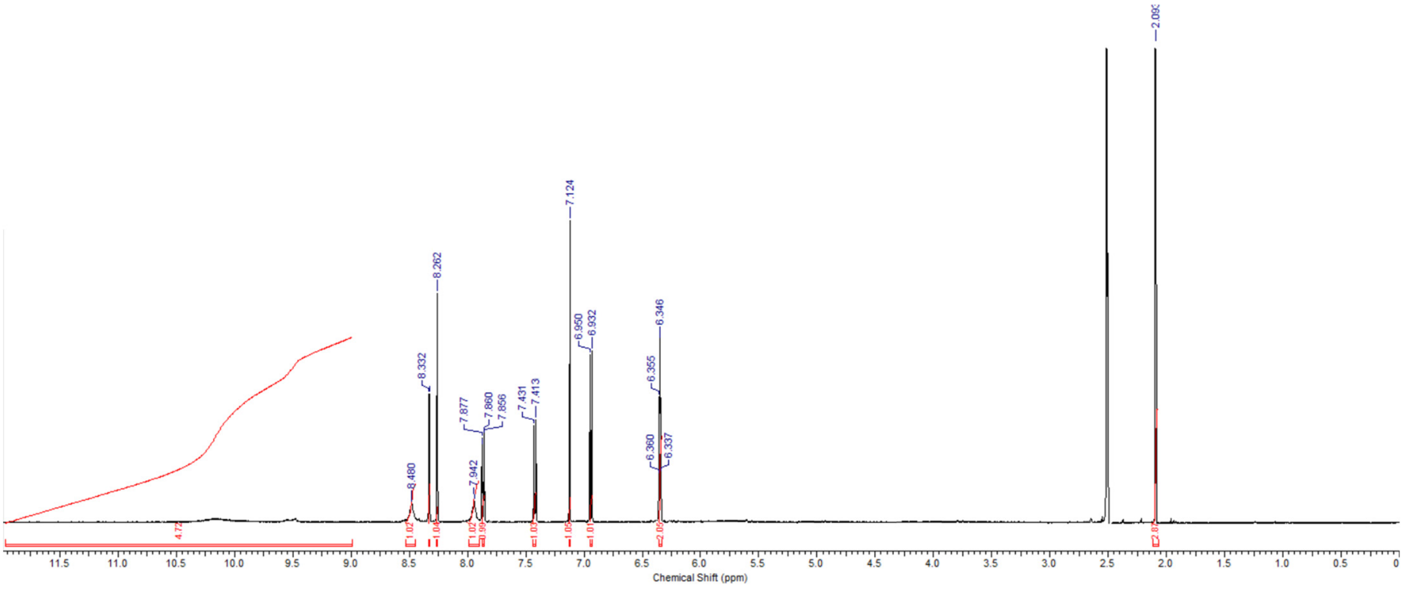

Figure S19. ^{1}H-NMR spectrum of compound **7b**.

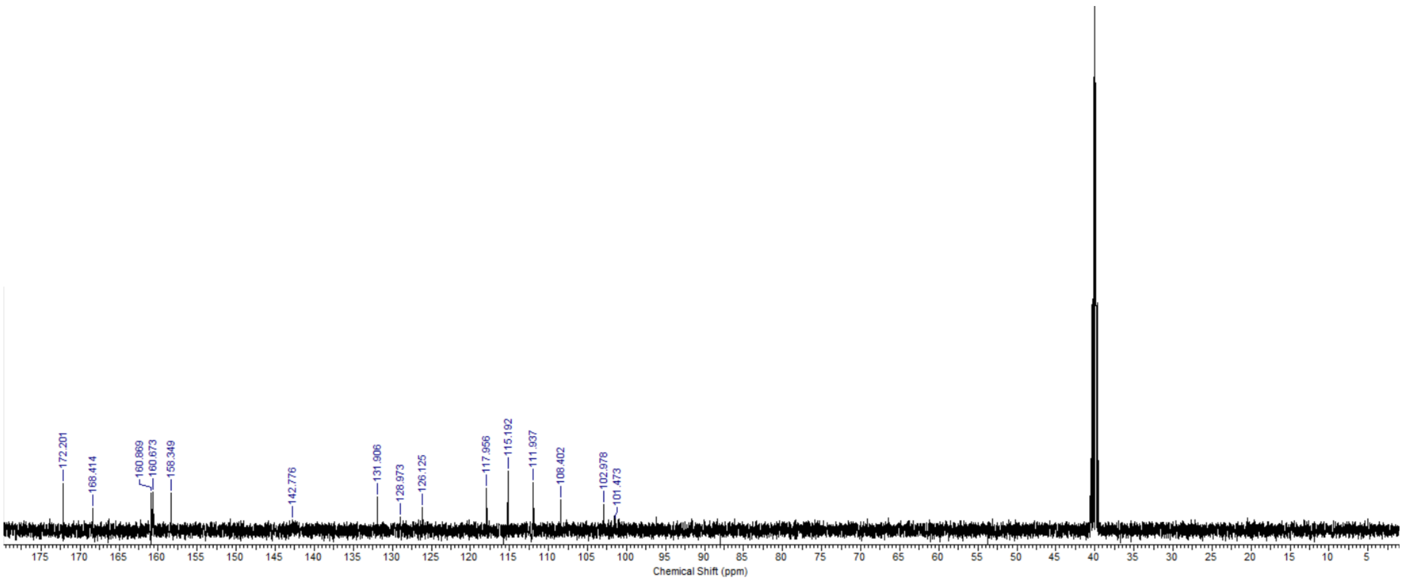

Figure S20. ^{13}C-NMR spectrum of compound **7b**.

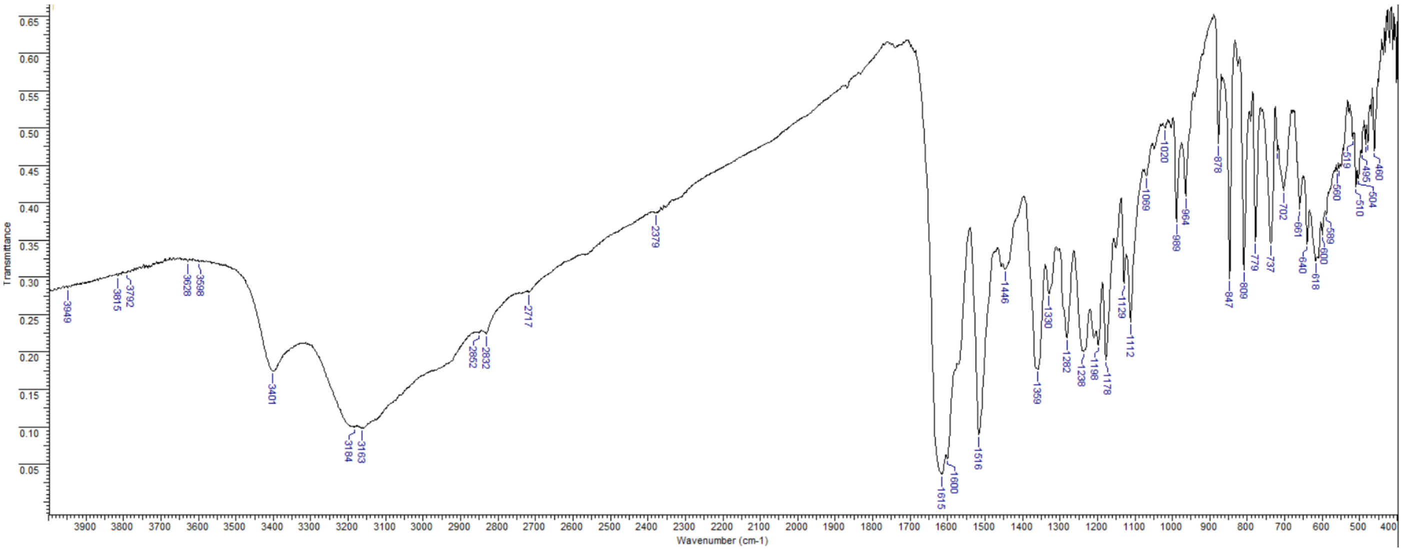

Figure S21. FTIR spectrum of compound **8a**.

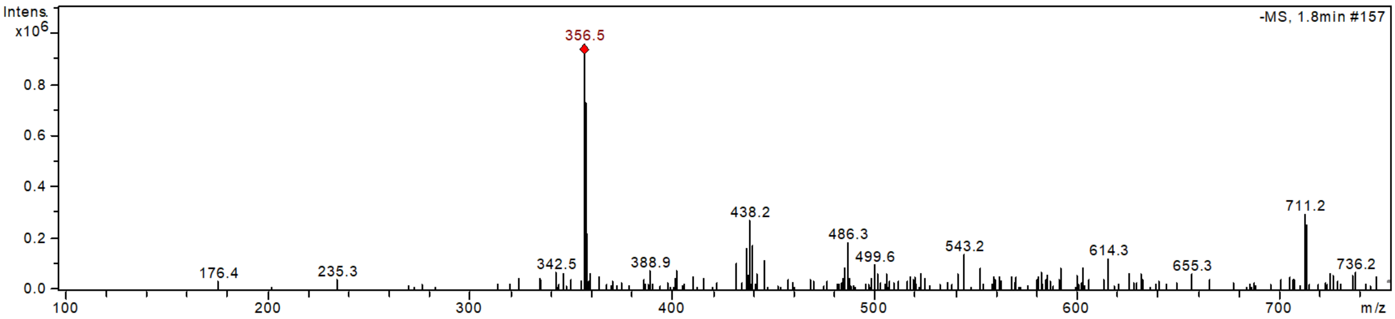

Figure S22. MS spectrum of compound **8a**.

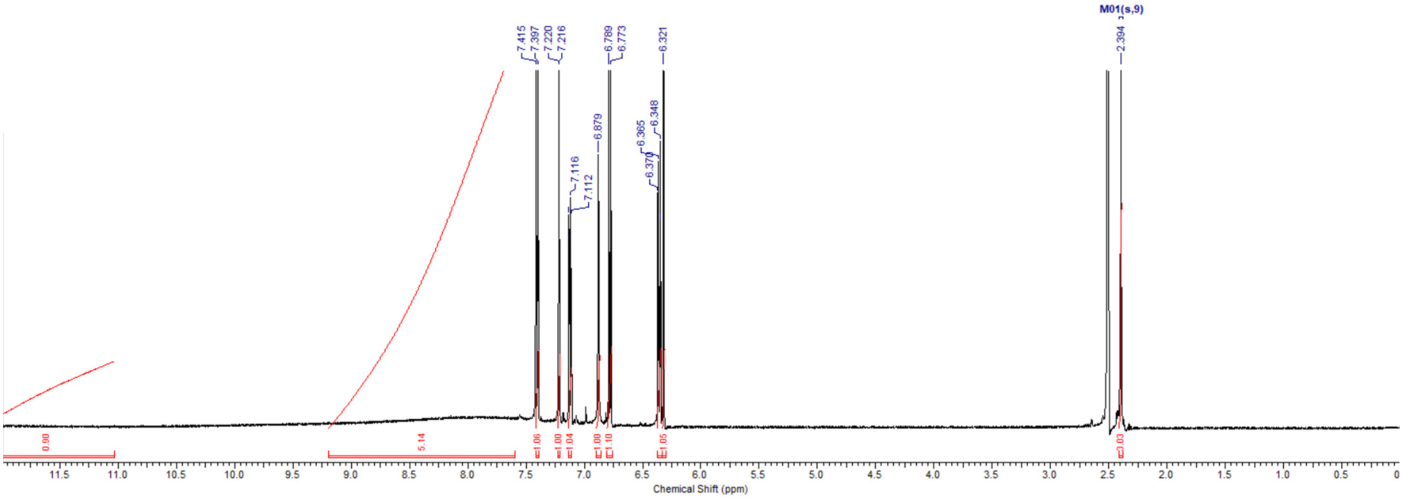

Figure S23. ^{1}H-NMR spectrum of compound **8a**.

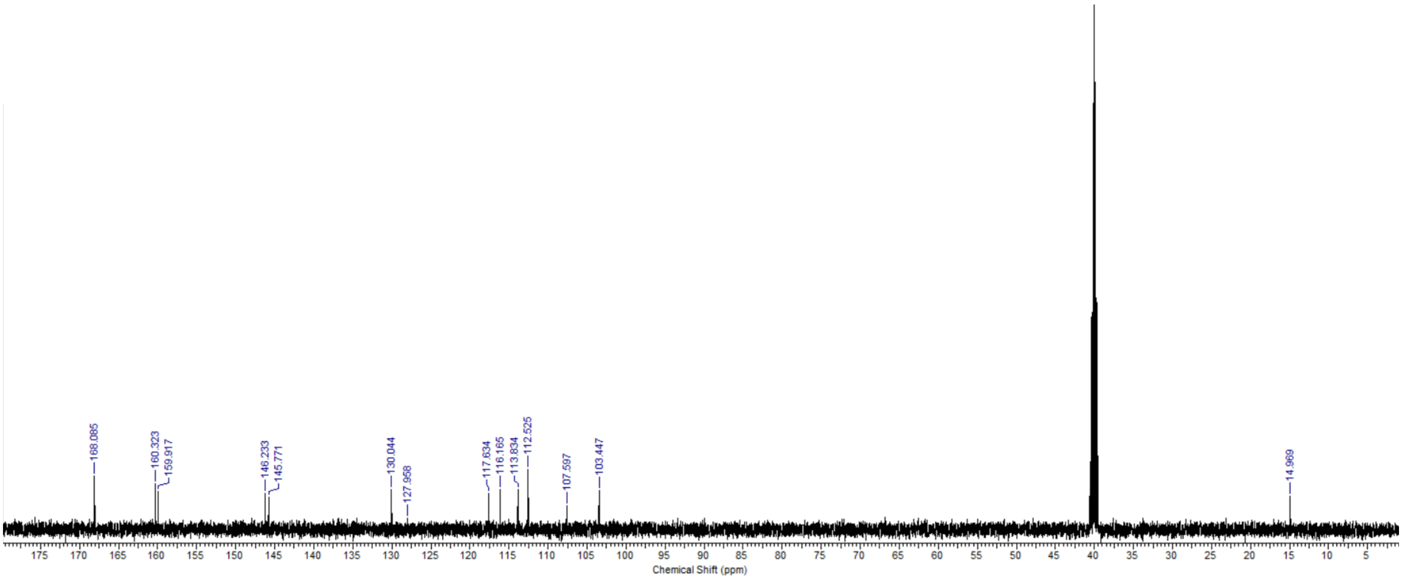

Figure S24. ^{13}C-NMR spectrum of compound **8a**.

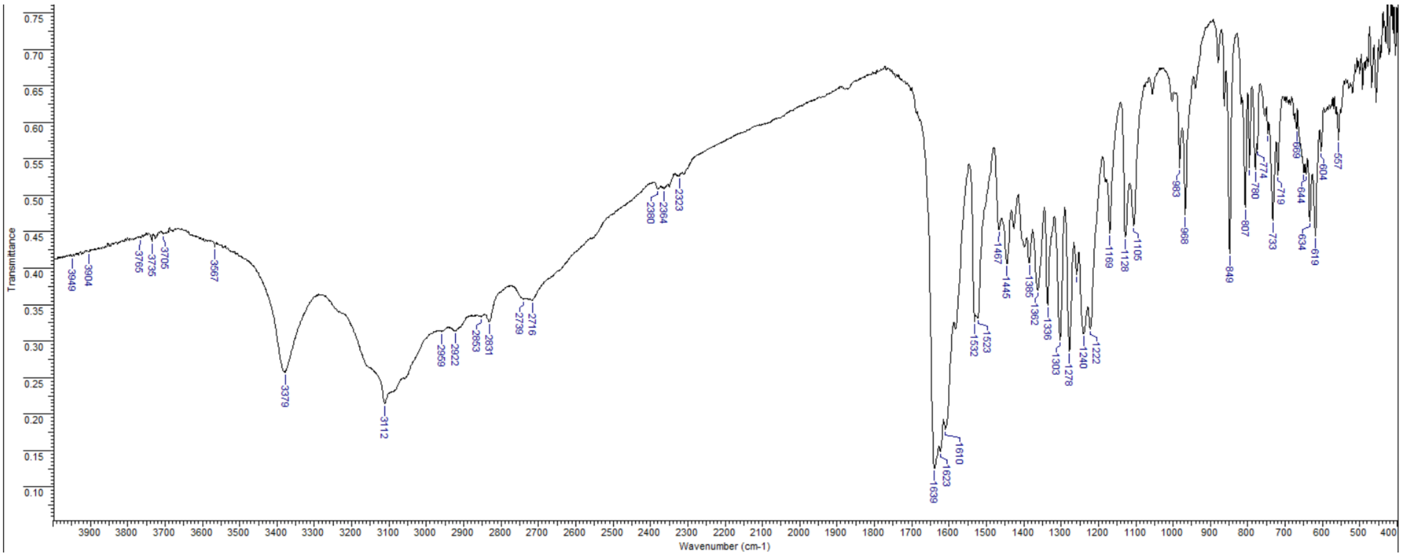

Figure S25. FTIR spectrum of compound **8b**.

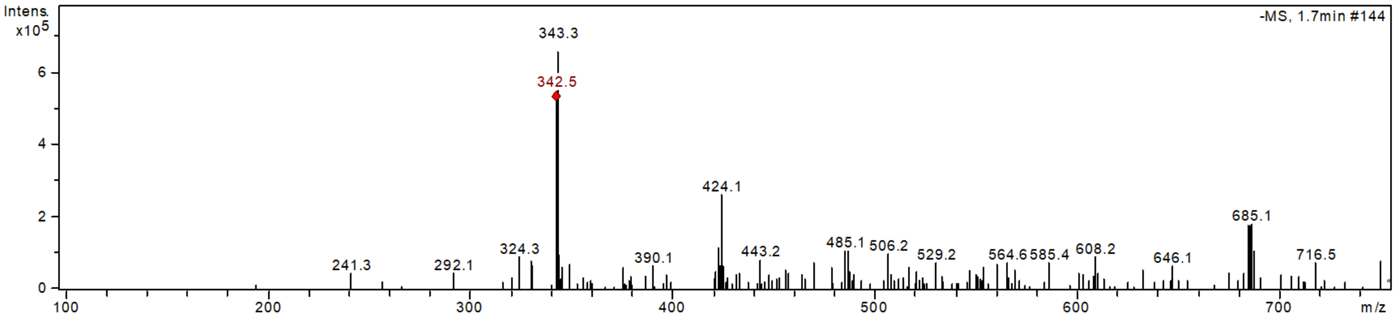

Figure S26. MS spectrum of compound **8b**.

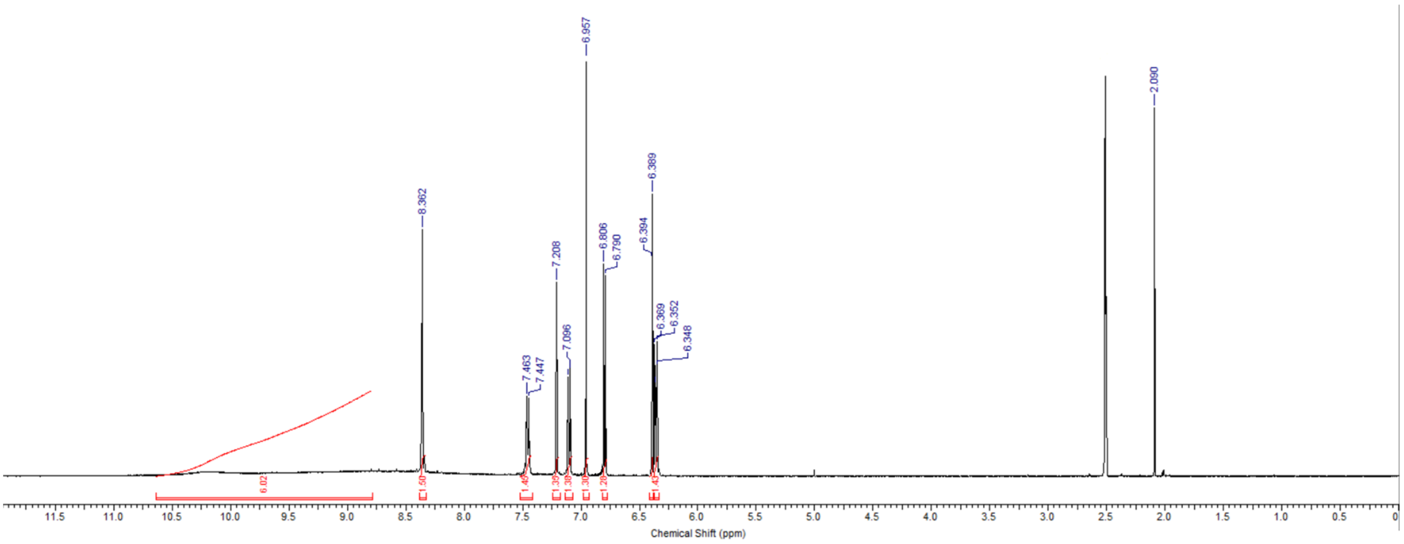

Figure S27. ^{1}H-NMR spectrum of compound **8b**.

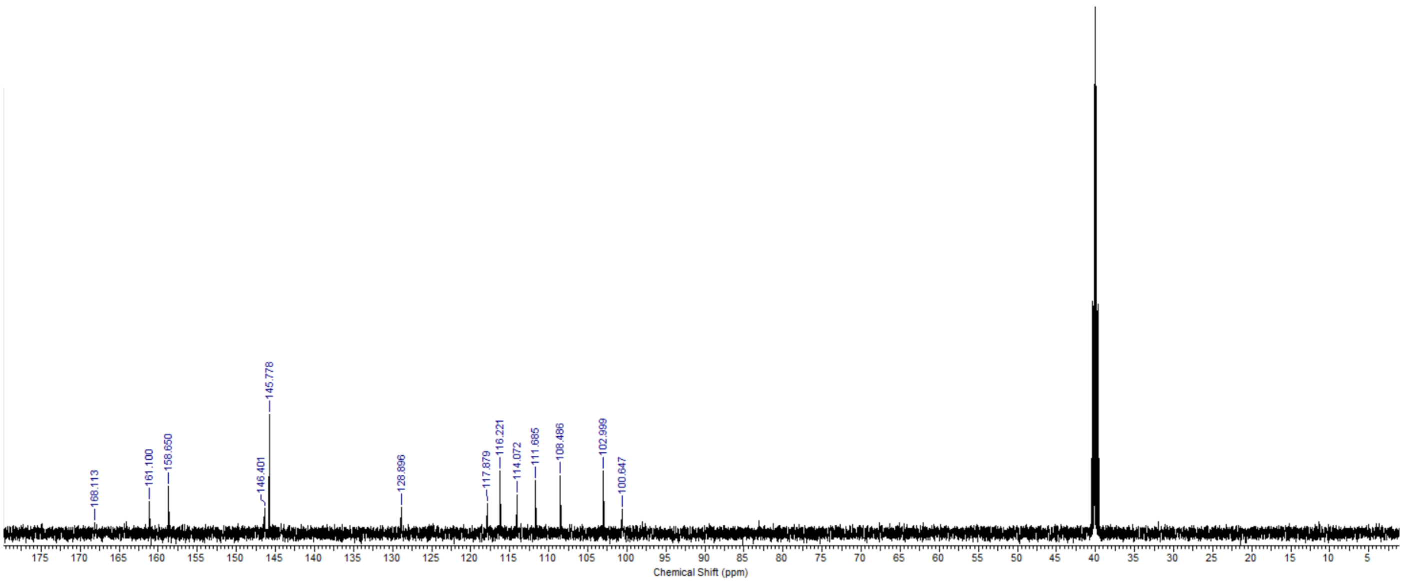

Figure S28. ^{13}C-NMR spectrum of compound **8b**.
